# Supplementary material for: K63-linked ubiquitination regulates RIPK1 kinase activity to prevent cell death during embryogenesis and inflammation
Source: Nat Commun. 2019 Sep 13;10:4157. doi: 10.1038/s41467-019-12033-8 (PMC6744441; doi:10.1038/s41467-019-12033-8)
Supplement: Supplementary file 1 — Supplementary information [file 41467_2019_12033_MOESM1_ESM.pdf]

Tang et al, K63-linked ubiquitination regulates RIPK1 kinase activity to prevent cell death during embryogenesis and inflammation

Supplementary Figures

Genotypes/ Embryo Stage	E9.5	E10.5	E11.5	E12.5	E13.5	E14.5
Ripk1$^{+/+}$	2	4	12	10	12	15
Ripk1$^{K376R/+}$	5	8	24	18	22	28
Ripk1$^{K376R/K376R}$	2	5	10	8	4	0
Total	9	17	46	36	38	43

Supplementary Figure 1. *Ripk1*$^{K376R/K376R}$ mice are embryonic lethal at E13.5

(**a**) Genotype analysis of offspring from *Ripk1*$^{K376R/+}$ intercrosses mice at different embryonic stage.

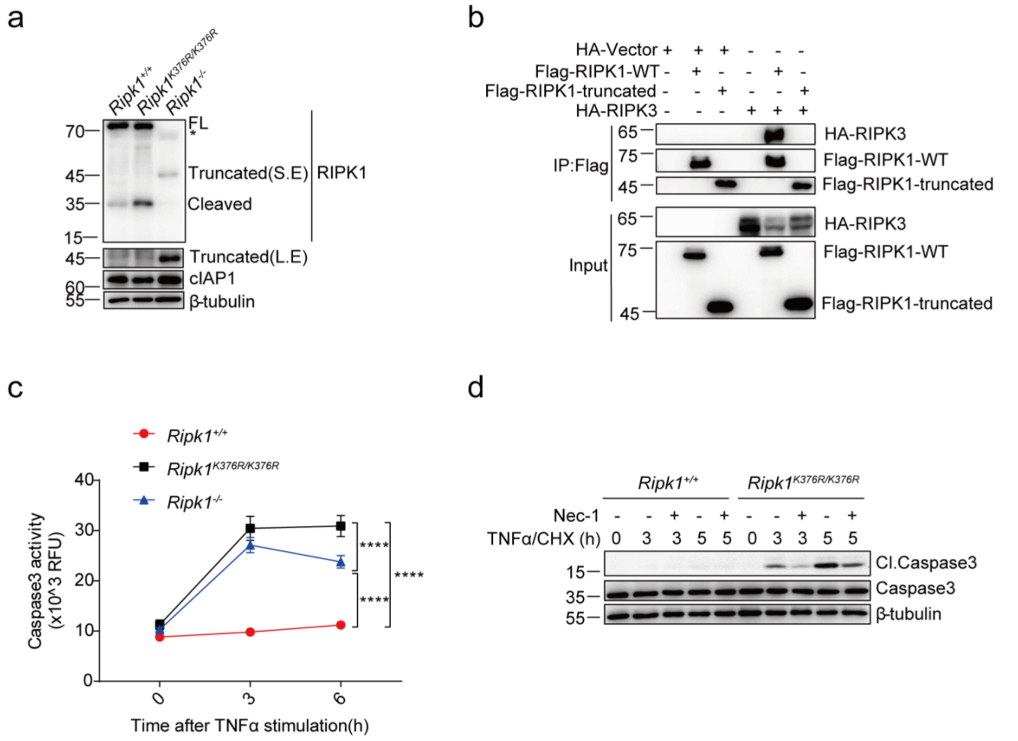

Supplementary Figure 2. *Ripk1*$^{K376R/K376R}$ mutation sensitizes cells to apoptosis and necroptosis

(**a**) Western-blotting detection of RIPK1 protein expression in primary MEFs from *Ripk1*$^{+/+}$, *Ripk1*$^{K376R/K376R}$ and *Ripk1*$^{-/-}$ mice (* refers to non-specific band). (**b**) Western-blotting analysis of immunoprecipitates using anti-Flag beads and total lysates of 293T cells transfected with the plasmids encoding Flag-RIPK1-WT, Flag-RIPK1-truncated and HA-RIPK3. (**c**) Caspase3 activity of *Ripk1*$^{+/+}$, *Ripk1*$^{K376R/K376R}$ and *Ripk1*$^{-/-}$ immortalized MEFs treated with TNFα (40ng/ml) for different time point were measured by DEVD-AMC fluorescence, the error bars

represent mean±s.e.m of data from three independent cell samples for each genotype. (**d**) *Ripk1*$^{+/+}$ and *Ripk1*$^{K376R/K376R}$ immortalized MEFs were treated with TNFα (10ng/ml) /CHX (10ug/ml) with or without pre-treatment of Necrostatin-1 for the indicated time, and the cell lysates were analyzed by western-blotting using the indicated antibodies. Statistical significance was determined using a two-tailed unpaired *t* test, ****P < 0.0001.

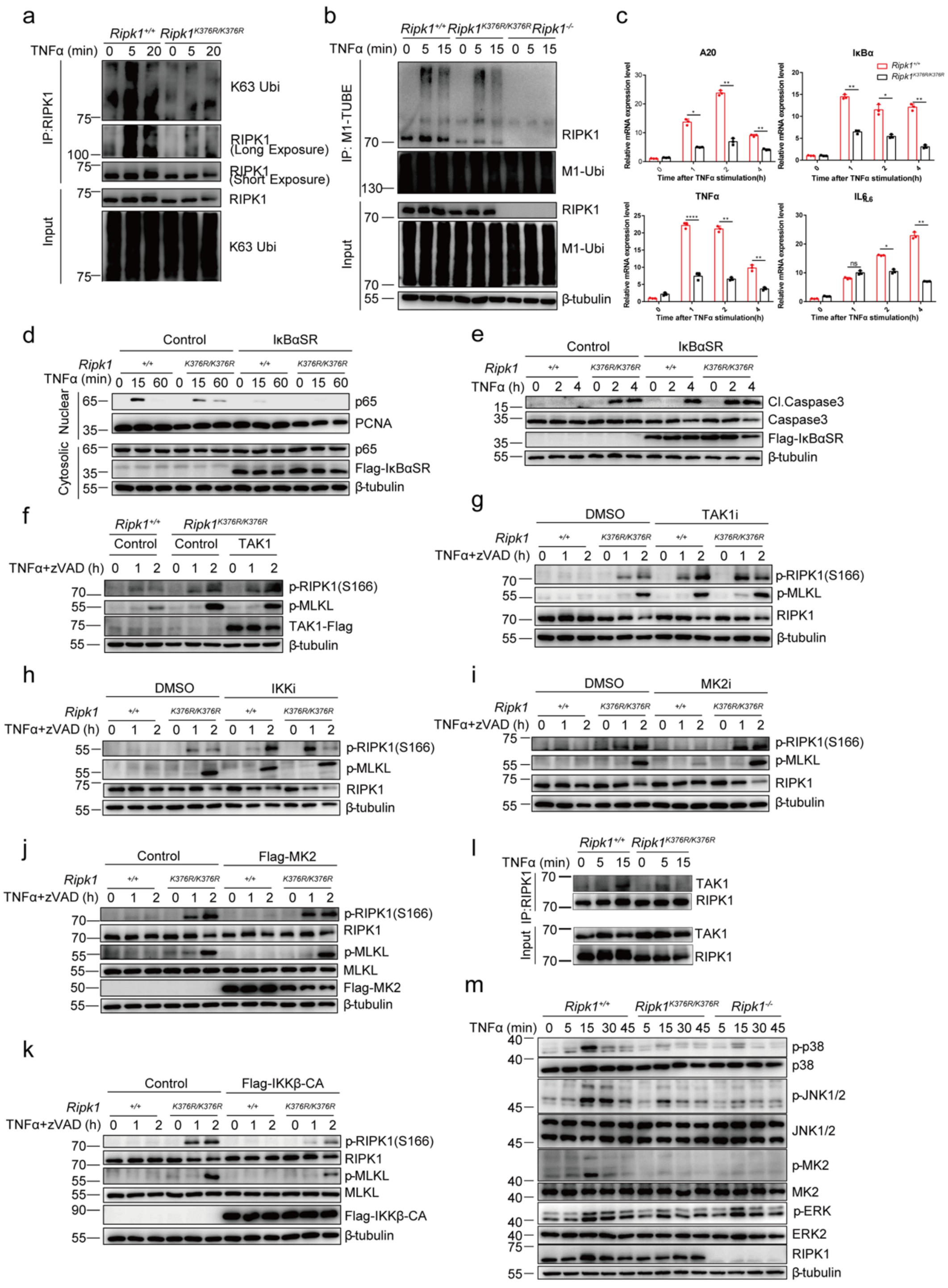

Supplementary Figure 3. *Ripk1*$^{K376R/K376R}$ mutation increases RIPK1 kinase activity

(**a**) Immunoprecipitation of RIPK1 antibody in *Ripk1*$^{+/+}$ and *Ripk1*$^{K376R/K376R}$ immortalized MEFs treated for the indicated time with TNFα (20 ng/ml). The immunocomplexes were

analyzed by western-blotting with antibody against K63-linked ubiquitination chains. (**b**) Immortalized *Ripk1*$^{+/+}$, *Ripk1*$^{K376R/K376R}$ and *Ripk1*$^{-/-}$ MEFs were treated for the indicated time with TNFα (20 ng/ml). The M1-ubiquitylated proteins were isolated by M1-TUBEs and analyzed by western-blotting. (**c**) RT-PCR analysis of NF-κB-targeting genes expression in *Ripk1*$^{+/+}$ or *Ripk1*$^{K376R/K376R}$ MEFs treated with TNFα (20 ng/ml) for the indicated time, the error bars represent mean±s.e.m of data from three independent cell samples for each genotype. (**d**) Nuclear extracts were collected from *Ripk1*$^{+/+}$ and *Ripk1*$^{K376R/K376R}$ MEFs treated with TNFα (20 ng/ml) at indicated time with or without stably expressed Flag-tagged IκBα-SR and analyzed by western-blotting with antibodies against p65 and PCNA. (**e**) *Ripk1*$^{+/+}$ and *Ripk1*$^{K376R/K376R}$ immortalized MEFs that stably expressed with the Flag-tagged IκBα-SR were stimulated with TNFα (40 ng/ml) for different periods of time. The cell lysates were analyzed by western-blotting with indicated antibodies. (**f,j,k**) *Ripk1*$^{+/+}$ and *Ripk1*$^{K376R/K376R}$ immortalized MEFs that expressed with the Flag-tagged TAK1(**f**) , Flag-tagged MK2(**j**) or Flag-IKKβ-CA(**k**) were stimulated with TNFα (20 ng/ml) plus zVAD.fmk (10 μM) for different periods of time. The cell lysates were analyzed by western-blotting with indicated antibodies. (**g-i**) *Ripk1*$^{+/+}$ and *Ripk1*$^{K376R/K376R}$ immortalized MEFs were stimulated with TNFα (20 ng/ml) plus zVAD.fmk (10 μM) for different periods of time with or without pre-treatment of TAK1 inhibitor(**g**), IKK inhibitor(**h**), MK2 inhibitor(**i**), The cell lysates wered analyzed by western-blotting with indicated antibodies. (**l**) Immunoprecipitation of RIPK1 antibody in *Ripk1*$^{+/+}$ and *Ripk1*$^{K376R/K376R}$ immortalized MEFs treated for the indicated time with TNFα (20 ng/ml). The immunocomplexes were analyzed by western-blotting with antibody against TAK1. (**m**) Immortalized *Ripk1*$^{+/+}$, *Ripk1*$^{K376R/K376R}$ and *Ripk1*$^{-/-}$ MEFs were treated for the indicated time with TNFα (20 ng/ml). The cell lysates were analyzed by western-blotting with indicated antibodies. Statistical significance was determined using a two-tailed unpaired *t* test, ns P > 0.05, *P < 0.05, **P < 0.01, ****P < 0.0001.

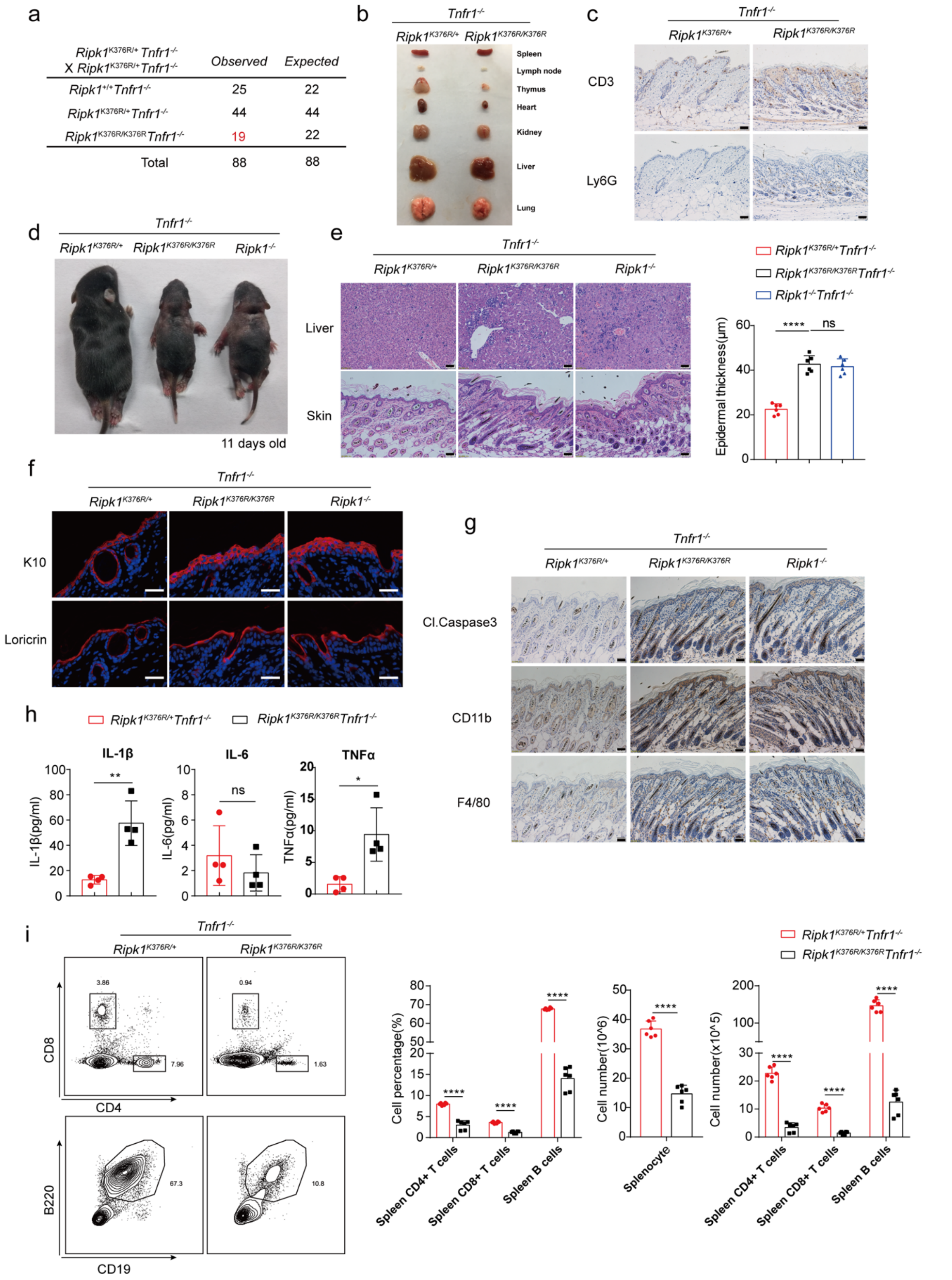

Supplementary Figure 4. TNFR1 deficiency partially delay the lethality of *Ripk1*$^{K376R/K376R}$ mice

(**a**) Statistical analysis of the expected and observed offspring mice (11-days-old) from the intercrosses of *Ripk1*$^{K376R/+}$*Tnfr1*$^{-/-}$ mice. (**b**) Representative macroscopic images of organs with

indicated genotypes at P11. (**c**) Immunohistochemical staining of CD3 and Ly6G in skin sections of *Ripk1*$^{K376R/K376R}$*Tnfr1*$^{-/-}$ and *Ripk1*$^{K376R/+}$*Tnfr1*$^{-/-}$ littermate mice at P11(scale bar,50 μm). (**d**) Representative macroscopic images of *Ripk1*$^{K376R/+}$*Tnfr1*$^{-/-}$, *Ripk1*$^{K376R/K376R}$*Tnfr1*$^{-/-}$ and *Ripk1*$^{-/-}$*Tnfr1*$^{-/-}$ mice at P11. (**e**) H&E staining of liver and skin sections of *Ripk1*$^{K376R/+}$*Tnfr1*$^{-/-}$, *Ripk1*$^{K376R/K376R}$*Tnfr1*$^{-/-}$ and *Ripk1*$^{-/-}$*Tnfr1*$^{-/-}$ mice at P11(scale bar,50 μm), and microscopic quantification of the epidermal thickness from H&E results (*Ripk1*$^{K376R/+}$*Tnfr1*$^{-/-}$ mice: n=6; *Ripk1*$^{K376R/K376R}$*Tnfr1*$^{-/-}$ mice: n=6; *Ripk1*$^{-/-}$*Tnfr1*$^{-/-}$ mice: n=6). (**f-g**) Immunofluorescence staining of Loricrin and K10 (scale bar,100 μm) (**f**) or immunohistochemical staining of F4/80, CD11b, and cleaved Caspase3(scale bar,50 μm) (**g**) in skin sections of *Ripk1*$^{K376R/+}$*Tnfr1*$^{-/-}$, *Ripk1*$^{K376R/K376R}$*Tnfr1*$^{-/-}$ and *Ripk1*$^{-/-}$*Tnfr1*$^{-/-}$ mice at P11. (**h**) Cytokines in lung homogenates were determined with the indicated genotypes at P11(*Ripk1*$^{K376R/+}$*Tnfr1*$^{-/-}$ mice: n=4; *Ripk1*$^{K376R/K376R}$*Tnfr1*$^{-/-}$ mice: n=4). (**i**) Flow cytometry and statistical results of CD4^{+}, CD8^{+} T cells and CD19^{+}B220^{+} B cells in spleen from *Ripk1*$^{K376R/K376R}$*Tnfr1*$^{-/-}$ and *Ripk1*$^{K376R/+}$*Tnfr1*$^{-/-}$ littermate mice at P11 (*Ripk1*$^{K376R/+}$*Tnfr1*$^{-/-}$ mice: n=6; *Ripk1*$^{K376R/K376R}$*Tnfr1*$^{-/-}$ mice: n=6). In (**e, h, i**), data are mean± s.e.m. Statistical significance was determined using a two-tailed unpaired *t* test, ns P > 0.05, *P < 0.05, **P < 0.01, ****P < 0.0001.

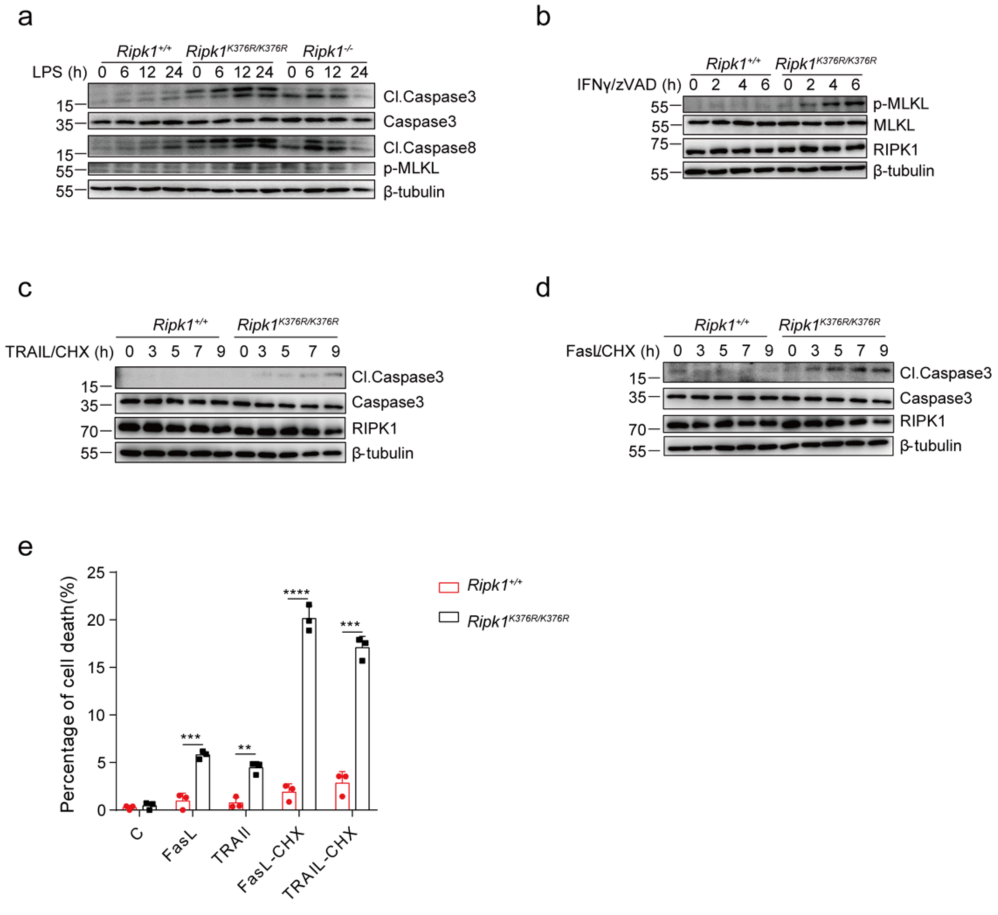

Supplementary Figure 5. Other signaling such as IFNs also contributes to lethality of *Ripk1*$^{K376R/K376R}$ mice

(**a-d**) *Ripk1*$^{+/+}$, *Ripk1*$^{K376R/K376R}$ and *Ripk1*$^{-/-}$ immortalized MEFs were treated with LPS (100 ng/ml) (**a**), IFNγ/zVAD (IFNγ: 10 μg/ml; zVAD.fmk: 20 μM) (**b**), TRAIL/CHX

(TRAIL:150ng/ml; CHX:10 ug/ml) (**c**) and FasL/CHX (FasL: 0.5ug/ml; CHX:10ug/ml) (**d**) for the indicated time. The cell lysates were analyzed by western-blotting using the indicated antibodies. (**e**) Cell death of *Ripk1*$^{+/+}$ and *Ripk1*$^{K376R/K376R}$ immortalized MEFs treated for 12h with different stimulators were measured by SytoxGreen positivity. FasL: 0.5ug/ml; TRAIL:150ng/ml; C: CHX (10ug/ml), the error bars represent mean±s.e.m of data from three independent cell samples for each genotype. Statistical significance was determined using a two-tailed unpaired *t* test, **P < 0.01, ***P < 0.01, ****P < 0.0001.

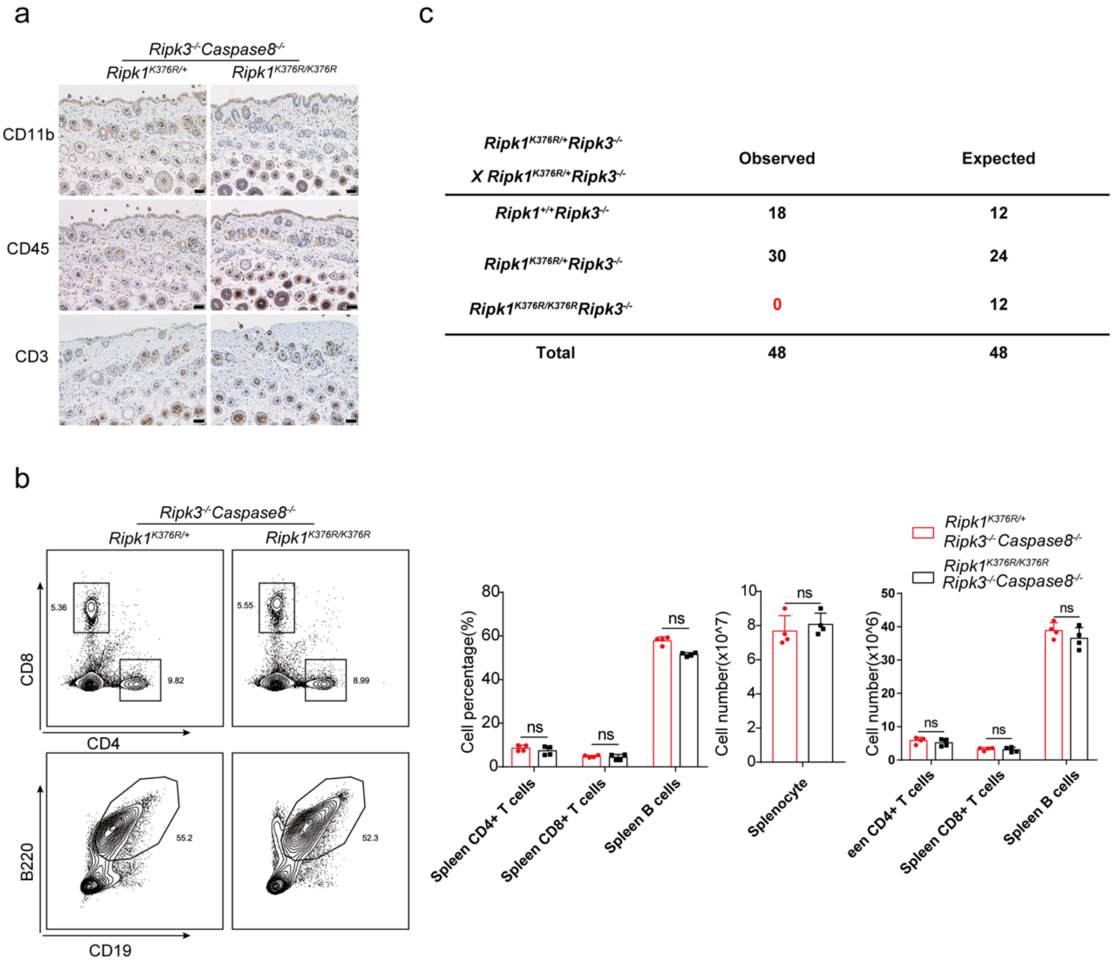

Ripk1K376R/+Ripk3-/- X Ripk1K376R/+Ripk3-/-	Observed	Expected
Ripk1+/+Ripk3-/-	18	12
Ripk1K376R/+Ripk3-/-	30	24
Ripk1K376R/K376RRipk3-/-	0	12
Total	48	48

Supplementary Figure 6. Co-deletion of RIPK3 and Caspase8 fully rescue the phenotype of *Ripk1*$^{K376R/K376R}$ mice

(**a**) Immunohistochemical staining of CD11b, CD45 and CD3 staining in skin sections of *Ripk1*$^{K376R/K376R}$*Ripk3*$^{-/-}$*Caspase8*$^{-/-}$ and *Ripk1*$^{K376R/+}$*Ripk3*$^{-/-}$*Caspase8*$^{-/-}$littermate mice at P40(scale bar,50 μm). (**b**) Flow cytometry and statistical results of CD4^{+}, CD8^{+} T cells and CD19^{+}B220^{+} B cells in spleen from *Ripk1*$^{K376R/K376R}$*Ripk3*$^{-/-}$*Caspase8*$^{-/-}$ and *Ripk1*$^{K376R/+}$*Ripk3*$^{-/-}$*Caspase8*$^{-/-}$ littermate mice at P40 (*Ripk1*$^{K376R/+}$*Ripk3*$^{-/-}$*Caspase8*$^{-/-}$ mice: n=4; *Ripk1*$^{K376R/K376R}$*Ripk3*$^{-/-}$ *Caspase8*$^{-/-}$ mice: n=4). (**c**) Statistical analysis of the expected and observed offspring mice (11-days-old) from the intercrosses of *Ripk1*$^{K376R/+}$*Ripk3*$^{-/-}$ mice. In (**b**), data are mean± s.e.m. Statistical significance was determined using a two-tailed unpaired *t* test, ns P > 0.05.

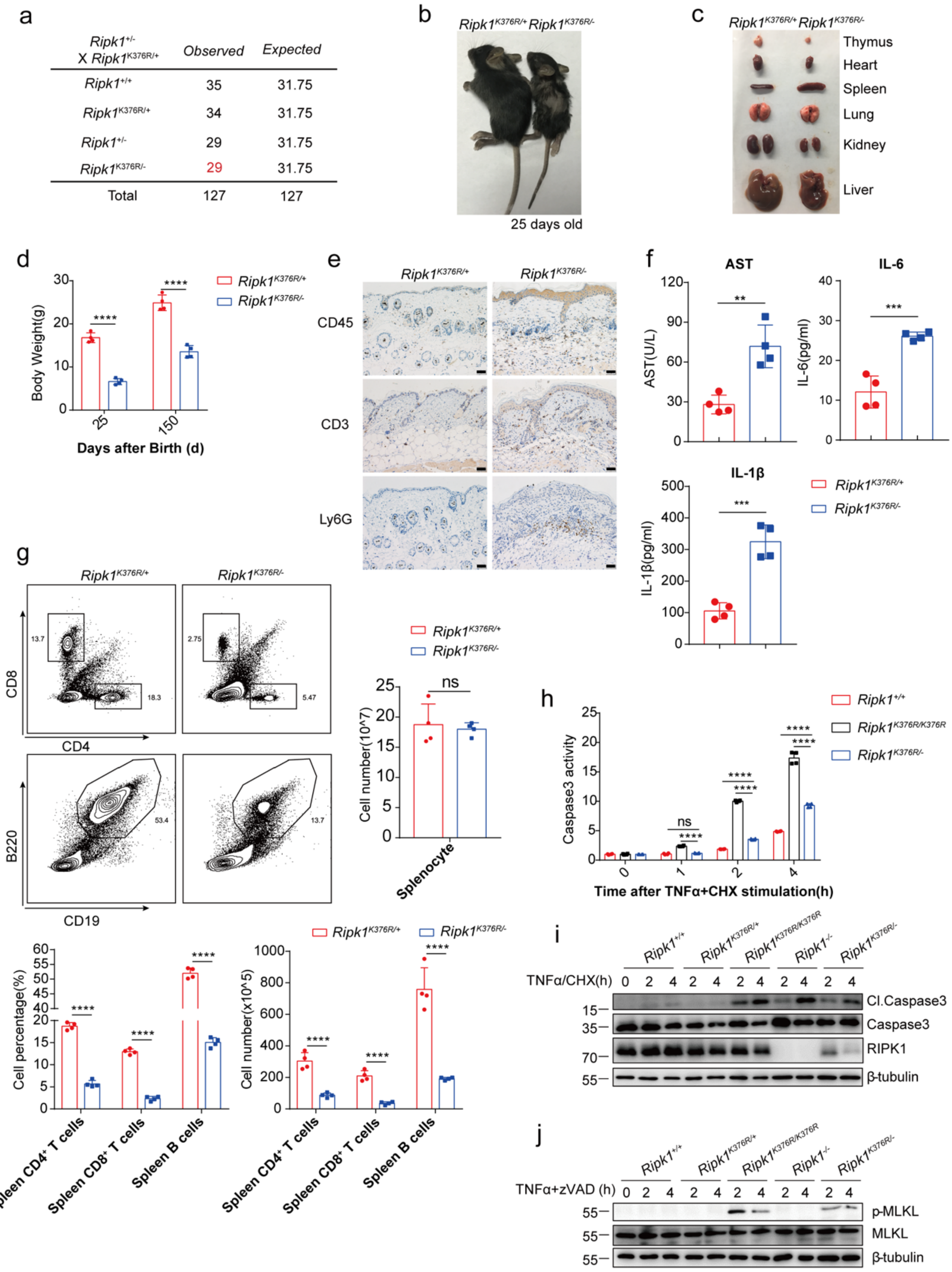

Supplementary Figure 7. ***Ripk1*** $^{K376R/-}$ **mice develops spontaneous inflammation**

(**a**) Statistical analysis of the expected and observed offspring mice (21-days-old) from the intercrosses of *Ripk1* $^{K376R/+}$ and *Ripk1* $^{+/-}$ mice. (**b**) Representative macroscopic images of *Ripk1* $^{K376R/-}$ and *Ripk1* $^{K376R/+}$ littermate mice at P25. (**c**) Representative macroscopic images of organs with indicated genotypes at P150. (**d**) Statistical analysis of body weight of *Ripk1* $^{K376R/+}$ and *Ripk1* $^{K376R/-}$ littermate mice at P25 and P150 (*Ripk1* $^{K376R/+}$ mice: n=4; *Ripk1* $^{K376R/-}$ mice: n=4). (**e**) Immunohistochemical staining of CD45, CD3 and Ly6G in skin sections of *Ripk1* $^{K376R/-}$ and

Ripk1$^{K376R/+}$ littermate mice at P150(scale bar,50 μm). (**f**) AST in blood and cytokines in liver homogenates were determined with the indicated genotypes at P150(*Ripk1*$^{K376R/+}$ mice: n=4; *Ripk1*$^{K376R/-}$ mice: n=4). (**g**) Flow cytometry and statistical results of $CD4^{+}$, $CD8^{+}$ T cells and $CD19^{+}B220^{+}$ B cells in spleen from *Ripk1*$^{K376R/+}$ and *Ripk1*$^{K376R/-}$ littermate mice at P150(*Ripk1*$^{K376R/+}$ mice: n=4; *Ripk1*$^{K376R/-}$ mice: n=4). (**h**) Caspase3 activity of *Ripk1*$^{+/+}$, *Ripk1*$^{K376R/-}$ and *Ripk1*$^{K376R/K376R}$ immortalized MEFs treated with TNFα/CHX (TNFα:20 ng/ml; CHX: 10 μg/ml) for the indicated time was measured by DEVD-AMC fluorescence, the error bars represent mean±s.e.m of data from three independent cell samples for each genotype. (**i-j**) *Ripk1*$^{+/+}$, *Ripk1*$^{K376R/+}$, *Ripk1*$^{K376R/K376R}$, *Ripk1*$^{-/-}$ and *Ripk1*$^{K376R/-}$ immortalized MEFs were treated with TNFα (20 ng/ml)/CHX (10 ug/ml) (**i**) and TNFα (20 ng/ml)/zVAD.fmk (10 μM) (**j**) for the indicated time, and the cell lysates were analyzed by western-blotting using the indicated antibodies. In (**d-h**), data are mean± s.e.m. Statistical significance was determined using a two-tailed unpaired *t* test, ns $P > 0.05$, $^{**}P < 0.01$, $^{***}P < 0.001$, $^{****}P < 0.0001$.

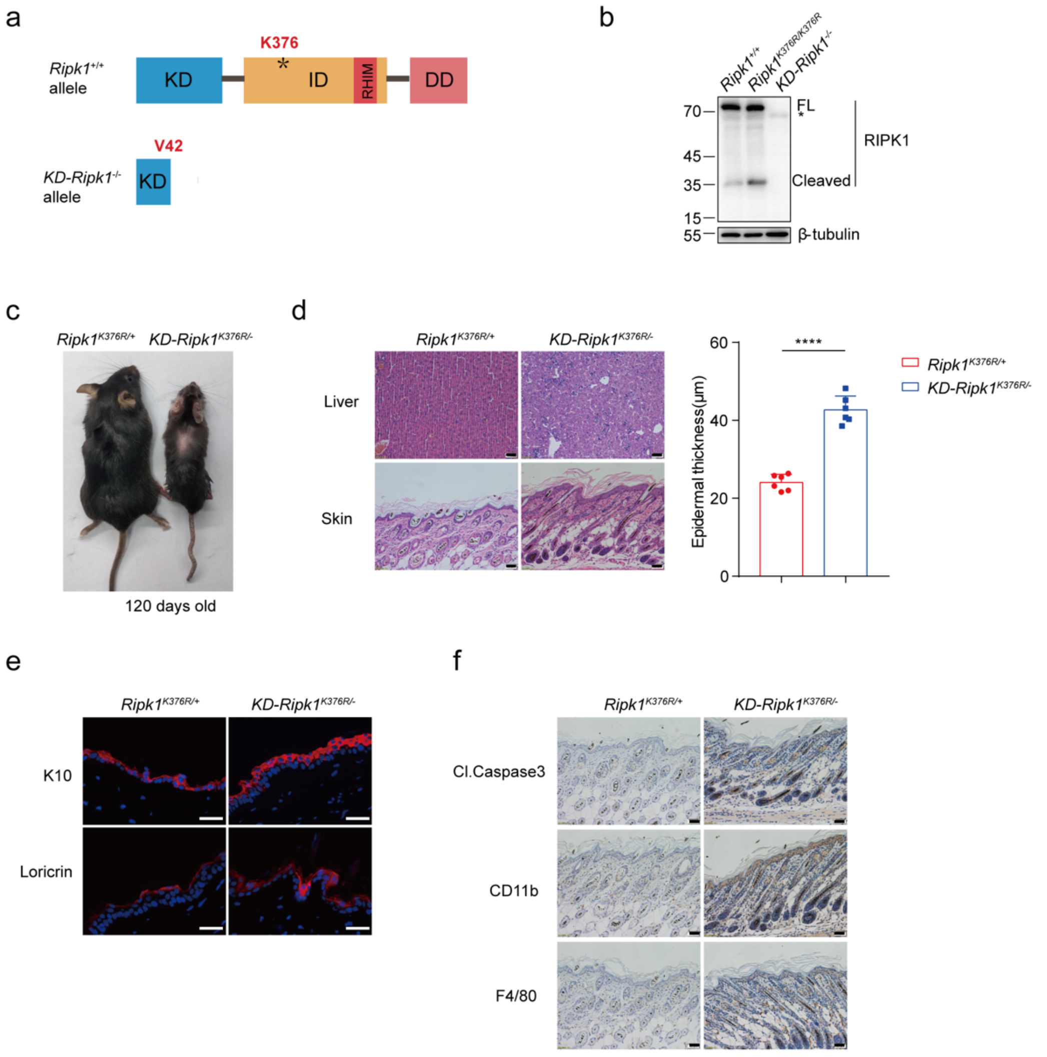

Supplementary Figure 8. *KD-Ripk1*$^{K376R/-}$ mice develops spontaneous inflammation

(**a**) Schematic overview of domain structure of *Ripk1*$^{K376R/K376R}$ and *KD-Ripk1*$^{-/-}$ mice. KD, kinase domain; ID, intermediate domain; DD, death domain; RHIM, RIP homotypic interaction motif. (**b**) Western-blotting detection of RIPK1 protein expression in primary MEFs from

Ripk1$^{+/+}$ and *KD-Ripk1*$^{-/-}$ mice (*refers to non-specific band). (**c**) Representative macroscopic images of *Ripk1*$^{K376R/+}$ and *KD-Ripk1*$^{K376R/-}$ littermate mice at P120. (**d**) H&E staining of liver and skin sections of *Ripk1*$^{K376R/+}$ and *KD-Ripk1*$^{K376R/-}$ littermate mice at P120 (scale bar,50 μm), and microscopic quantification of the epidermal thickness from H&E results (*Ripk1*$^{K376R/+}$ mice: n=4; *KD-Ripk1*$^{K376R/-}$ mice: n=4). (**e-f**) Immunofluorescence staining of Loricrin and K10 (scale bar,100 μm) (**e**) or immunohistochemical staining of F4/80, CD11b, and cleaved Caspase3(scale bar,50 μm) (**f**) in skin sections of *Ripk1*$^{K376R/+}$ and *KD-Ripk1*$^{K376R/-}$ littermate mice at P120. In (**d**), data are mean± s.e.m. Statistical significance was determined using a two-tailed unpaired *t* test, ****P < 0.0001.

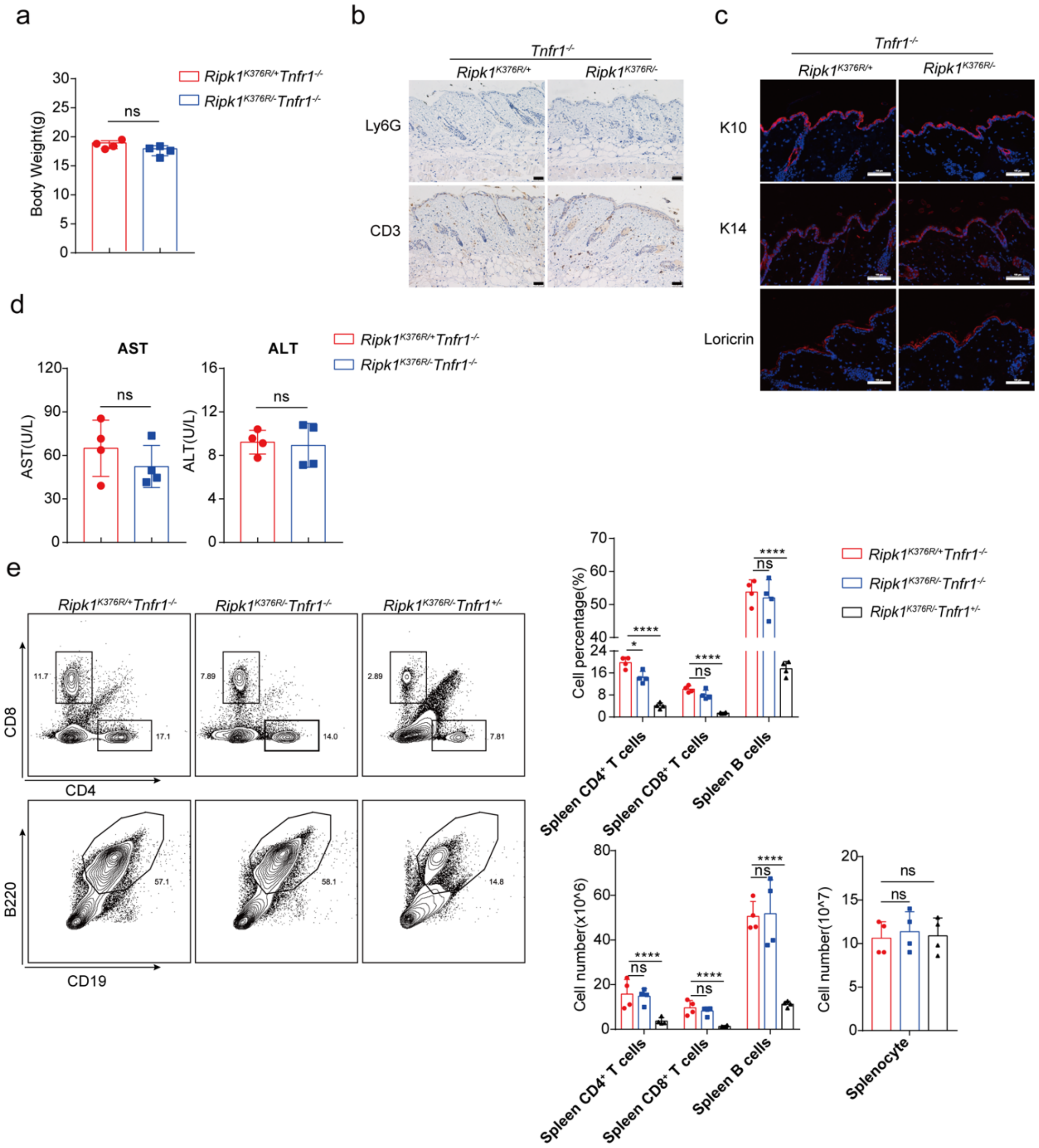

Supplementary Figure 9. TNFR1 deficiency suppress inflammation in *Ripk1*$^{K376R/-}$ mice

(**a**) Statistical analysis of body weight of *Ripk1*$^{K376R/-}$*Tnfr1*$^{-/-}$ and *Ripk1*$^{K376R/+}$*Tnfr1*$^{-/-}$ littermate mice at P40 (*Ripk1*$^{K376R/+}$*Tnfr1*$^{-/-}$ mice: n=4; *Ripk1*$^{K376R/-}$*Tnfr1*$^{-/-}$ mice: n=4). (**b**) Immunohistochemical staining of CD3 and Ly6G in skin sections of *Ripk1*$^{K376R/-}$*Tnfr1*$^{-/-}$ and *Ripk1*$^{K376R/+}$*Tnfr1*$^{-/-}$

littermate mice at P40(scale bar,50 μm). (**c**) Immunofluorescence staining of Loricrin, K10, and K14 in skin sections of *Ripk1*$^{K376R/-}$*Tnfr1*$^{-/-}$ and *Ripk1*$^{K376R/+}$*Tnfr1*$^{-/-}$ littermate mice at P40(scale bar,100 μm). (**d**) AST and ALT in blood were determined with the indicated genotypes at P40(*Ripk1*$^{K376R/+}$*Tnfr1*$^{-/-}$ mice: n=4; *Ripk1*$^{K376R/-}$*Tnfr1*$^{-/-}$ mice: n=4). (**e**) Flow cytometry and statistical results of CD4^{+}, CD8^{+} T cells and CD19^{+}B220^{+} B cells in spleen from *Ripk1*$^{K376R/-}$*Tnfr1*$^{-/-}$, *Ripk1*$^{K376R/-}$*Tnfr1*$^{+/-}$ and *Ripk1*$^{K376R/+}$*Tnfr1*$^{-/-}$ littermate mice at P150(*Ripk1*$^{K376R/+}$*Tnfr1*$^{-/-}$ mice: n=4; *Ripk1*$^{K376R/-}$*Tnfr1*$^{+/-}$ mice: n=4; *Ripk1*$^{K376R/-}$*Tnfr1*$^{-/-}$ mice: n=4). In (**a, d, e**), data are mean± s.e.m. Statistical significance was determined using a two-tailed unpaired *t* test, ns $P > 0.05$, $^{*}P < 0.05$, $^{**}P < 0.01$, $^{****}P < 0.0001$.

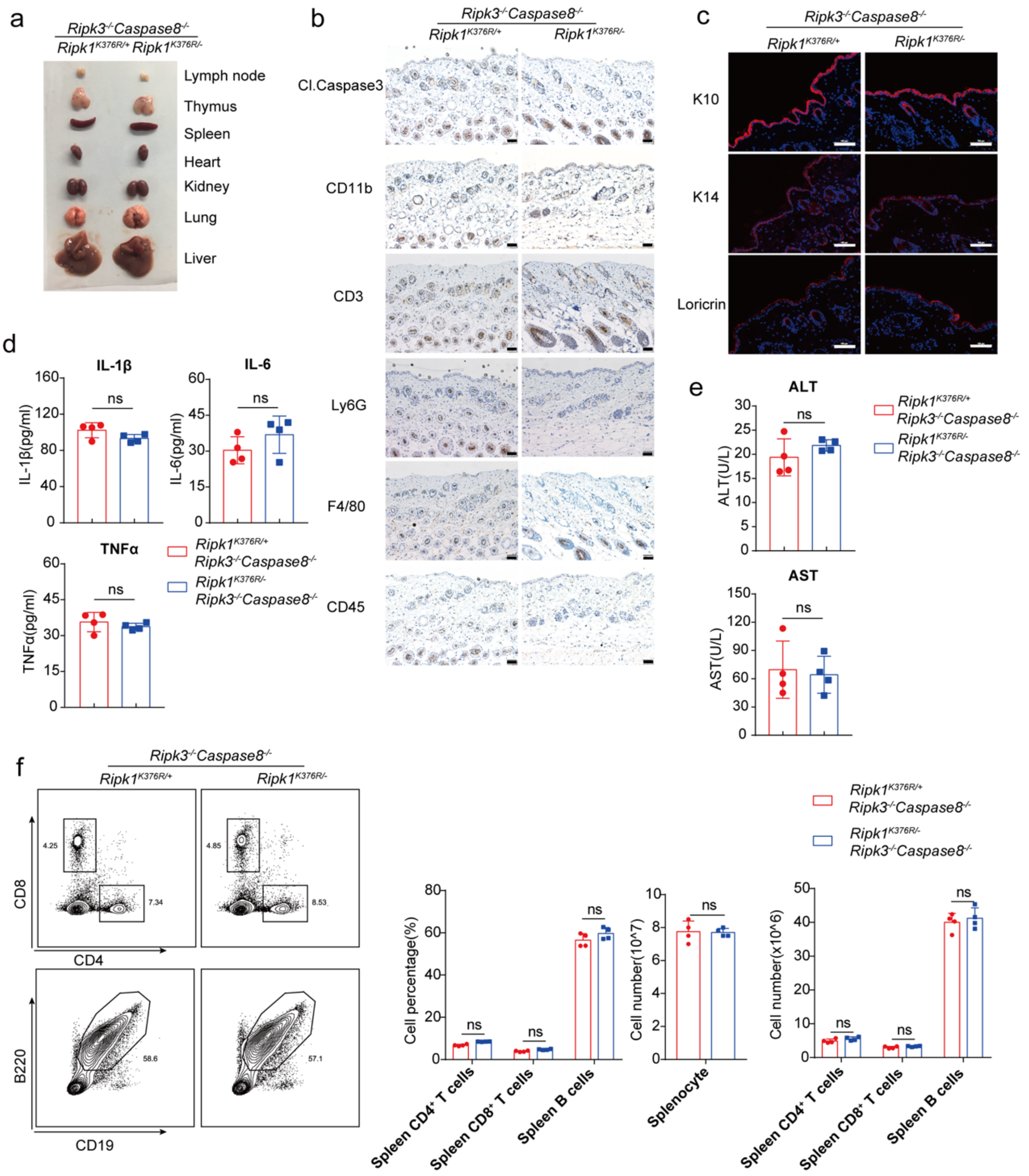

Supplementary Figure 10. Co-deletion of RIPK3 and Caspase8 suppress inflammation in *Ripk1*$^{K376R/-}$ mice

(**a**) Representative macroscopic images of organs with indicated genotypes at P40. (**b**) Immunohistochemical staining of cleaved Caspase3, CD11b, CD3, Ly6G, F4/80 and CD45 in skin sections of *Ripk1*$^{K376R/-}$*Ripk3*$^{-/-}$ *Caspase8*$^{-/-}$ and *Ripk1*$^{K376R/+}$*Ripk3*$^{-/-}$ *Caspase8*$^{-/-}$ littermate mice at P40(scale bar,50 μm). (**c**) Immunofluorescence staining of Loricrin, K10, and K14 in skin sections of *Ripk1*$^{K376R/-}$*Ripk3*$^{-/-}$ *Caspase8*$^{-/-}$ and *Ripk1*$^{K376R/+}$*Ripk3*$^{-/-}$ *Caspase8*$^{-/-}$ littermate mice at P40(scale bar,100 μm). (**d**) Cytokines in liver homogenates were determined with the indicated genotypes at P40 (*Ripk1*$^{K376R/+}$*Ripk3*$^{-/-}$*Caspase8*$^{-/-}$ mice: n=4; *Ripk1*$^{K376R/-}$*Ripk3*$^{-/-}$*Caspase8*$^{-/-}$ mice: n=4). (**e**) AST and ALT in blood were determined with the indicated genotypes at P40(*Ripk1*$^{K376R/+}$*Ripk3*$^{-/-}$*Caspase8*$^{-/-}$ mice: n=4; *Ripk1*$^{K376R/-}$*Ripk3*$^{-/-}$*Caspase8*$^{-/-}$ mice: n=4). (**f**) Flow cytometry and statistical results of CD4^{+}, CD8^{+} T cells and CD19^{+}B220^{+} B cells in spleen from *Ripk1*$^{K376R/-}$*Ripk3*$^{-/-}$ *Caspase8*$^{-/-}$ and *Ripk1*$^{K376R/+}$*Ripk3*$^{-/-}$ *Caspase8*$^{-/-}$ littermate mice at P40(*Ripk1*$^{K376R/+}$*Ripk3*$^{-/-}$*Caspase8*$^{-/-}$ mice: n=4; *Ripk1*$^{K376R/-}$*Ripk3*$^{-/-}$*Caspase8*$^{-/-}$ mice: n=4). In (**d, e, f**), data are mean± s.e.m. Statistical significance was determined using a two-tailed unpaired *t* test, ns P > 0.05.

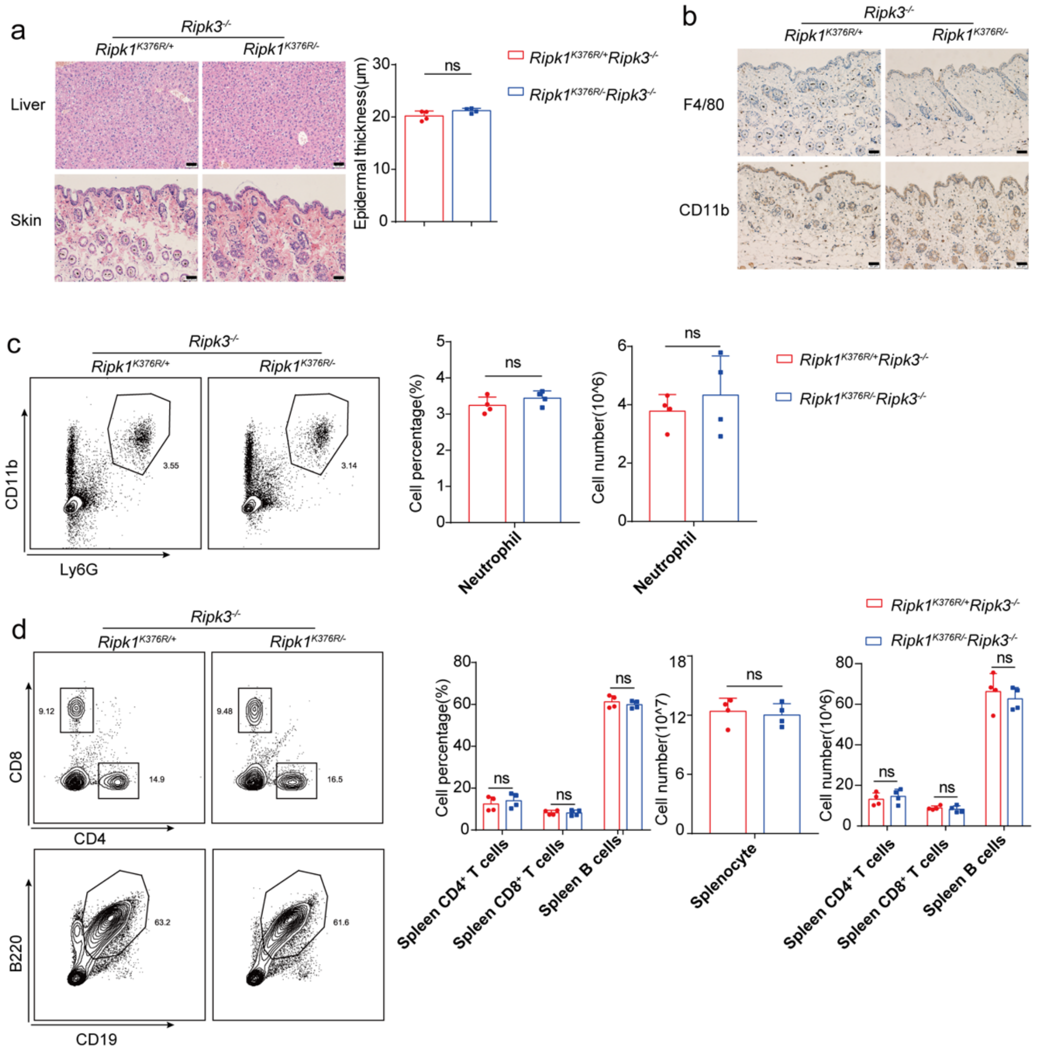

Supplementary Figure 11. RIPK3 deletion suppress inflammation in *Ripk1*$^{K376R/-}$ mice

(**a**) H&E staining of liver and skin sections of *Ripk1*$^{K376R/-}$*Ripk3*$^{-/-}$ and *Ripk1*$^{K376R/+}$*Ripk3*$^{-/-}$ littermate mice at P40(scale bar,50 μm), and microscopic quantification of the epidermal

thickness from H&E results (*Ripk1*$^{K376R/+}$*Ripk3*$^{-/-}$ mice: n=4; *Ripk1*$^{K376R/-}$*Ripk3*$^{-/-}$ mice: n=4). (**b**) Immunohistochemical staining of CD11b and F4/80 in skin sections of *Ripk1*$^{K376R/-}$*Ripk3*$^{-/-}$ and *Ripk1*$^{K376R/+}$*Ripk3*$^{-/-}$ littermate mice at P40(scale bar,50 μm). (**c**) Flow cytometry and statistical results of splenocytes stained with Ly6G and CD11b from *Ripk1*$^{K376R/-}$*Ripk3*$^{-/-}$ and *Ripk1*$^{K376R/+}$*Ripk3*$^{-/-}$ littermate mice at P40 (*Ripk1*$^{K376R/+}$*Ripk3*$^{-/-}$ mice: n=4; *Ripk1*$^{K376R/-}$*Ripk3*$^{-/-}$ mice: n=4). CD11b^{+}Ly6G^{+} cells were identified as neutrophils. (**d**) Flow cytometry and statistical results of CD4^{+}, CD8^{+} T cells and CD19^{+}B220^{+} B cells in spleen from *Ripk1*$^{K376R/-}$*Ripk3*$^{-/-}$ and *Ripk1*$^{K376R/+}$*Ripk3*$^{-/-}$ littermate mice at P40(*Ripk1*$^{K376R/+}$*Ripk3*$^{-/-}$ mice: n=4; *Ripk1*$^{K376R/-}$*Ripk3*$^{-/-}$ mice: n=4). In (**a, c, d**), data are mean± s.e.m. Statistical significance was determined using a two-tailed unpaired *t* test, ns $P > 0.05$.

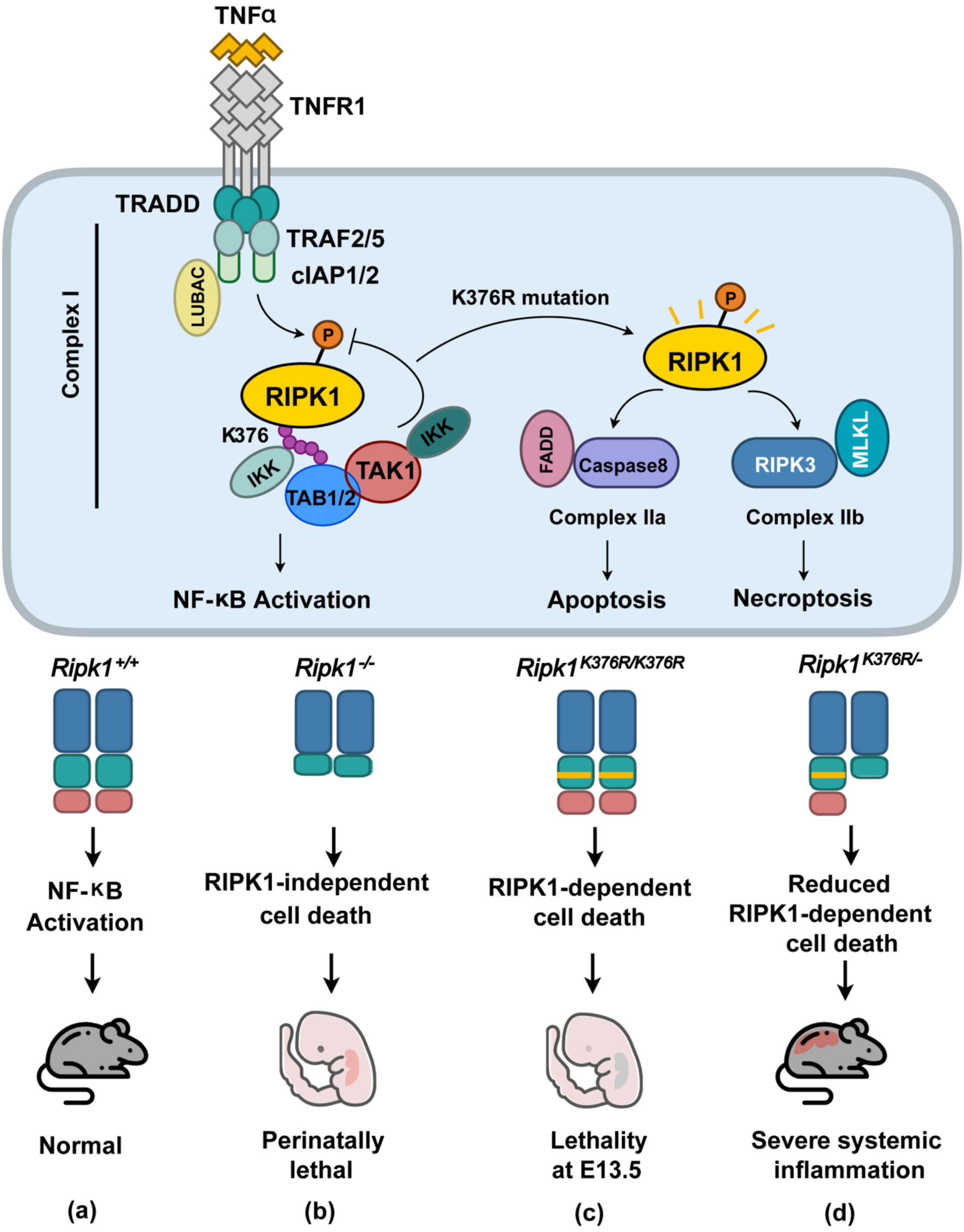

Supplementary Figure 12. Proposed model that K63-linked ubiquitination of RIPK1 on K376 regulating TNFα-induced signaling

(a) In normal condition, K63-linked ubiquitination of RIPK1 on K376 can recruit TAK1/IKK to suppress RIPK1 kinase activity, and also recruit IKK complex to promote NF-κB activation. These two signaling can both contribute to survival of *Ripk1*$^{+/+}$ mice. (b) Deficiency of RIPK1 can induce RIPK1-independent cell death, resulting in perinatally lethal of *Ripk1*$^{-/-}$ mice. (c) K376R mutation can enhance RIPK1 kinase activity to promote RIPK1-dependent cell death, resulting in embryonic lethality of *Ripk1*$^{K376R/K376R}$ mice at E13.5. (d) In *Ripk1*$^{K376R/-}$ cells,

RIPK1 kinase activity is relatively decreased comparing to *Ripk*1$^{K376R/K376R}$ cells, resulting in reduced RIPK1-denpendent cell death, and *Ripk*1$^{K376R/-}$ could survive but develop severe systemic inflammation.

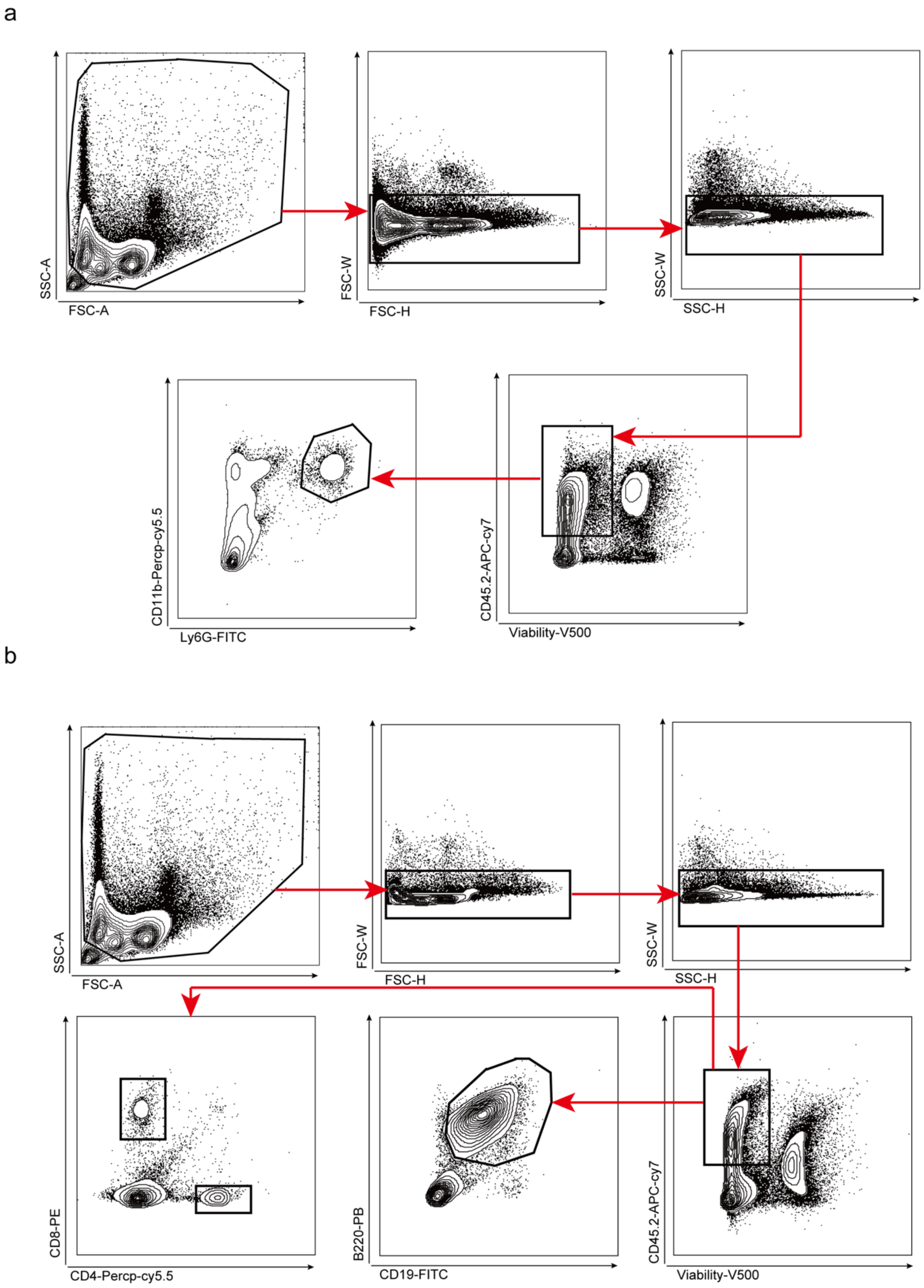

Supplementary Figure 13. Hematopoietic gating strategy

(a) For characterization of spleen neutrophils, the living cell fractions gated from preliminary FSC/SSC gates could be further divided into CD45+CD11b+Ly6G+ neutrophils. The

antibodies and fluorochrome used described as "Antibodies and reagents" in the methods.
(b) For characterization of spleen T cells, B cells, the living cell fractions gated from preliminary FSC/SSC gates could be further divided into three cell types: CD45+CD4+ T cells, CD45+CD8+ T cells, CD45+CD19+B220+ B cells. The antibodies and fluorochrome used described as "Antibodies and reagents" in the methods.

Supplementary Table

Table 1. Primer sequences for generation of *Ripk1*$^{K376R/K376R}$ and *KD-Ripk1*$^{-/-}$ mice.

sgRNA(*Ripk1*$^{K376R/K376R}$)	tgtgcaggctaagctgcaag
Donor(*Ripk1*$^{K376R/K376R}$)	gtcctggttttcttcctccccagagtacccacaggacgagaatgatcgcagtgtgcaggccagactacag gaagaagccagctatcatgcttttggaatatttgcagagaaacagacaaa
sgRNA(*KD-Ripk1*$^{-/-}$)	gccctgtgtatactttttc

Table 2. The primer sequences for genotyping-PCR.

Gene	Forward Primer (5'--3')	Reverse Primer (5'--3')
	**Primer sequences for genotyping-PCR**	
RIPK1 K376R WT	tgcaggctaagctgcaagag	gtgctgggatcagaatgacc
RIPK1 K376R KI	tgcaggccagactacaggaa	gtgctgggatcagaatgacc
RIPK1 ID-KO	ttcctccccagagtacccac	gtgctgggatcagaatgacc
RIPK1 KD-KO	gtggagtacaagctagcctcagac	ctgtgtttagccacacagatg

Table 3. The primer sequences for qRT-PCR.

Gene	Forward Primer (5'--3')	Reverse Primer (5'--3')
	**Primer sequences for qRT-PCR**	
GAPDH	AACAGCAACTCCCACTCTTC	CCTGTTGCTGTAGCCGTATT
IL6	TCCATCCAGTTGCCTTCTTG	GGTCTGTTGGGAGTGGTATC
TNFα	CTACCTTGTTGCCTCCTCTTT	GAGCAGAGGTTCAGTGATGTAG
IκBα	TGAAGGACGAGGAGTACGAGC	TTCGTGGATGATTGCCAAGTG
CXCL1	CTGGGATTCACCTCAAGAACATC	CAGGGTCAAGGCAAGCCTC

CXCL2	CCAACCACCAGGCTACAGG	GCGTCACACTCAAGCTCTG
IFNβ	CAGCTCCAAGAAAGGACGAAC	GGCAGTGTAACTCTTCTGCAT
IFNγ	CTTTGGACCCTCTGACTTGAG	TCAATGACTGTGCCGTGG
A20	ACAGTGGACCTGGTAAGAAAACA	CCTCCGTGACTGATGACAAGAT
