# Supplementary material for: K63-linked ubiquitination regulates RIPK1 kinase activity to prevent cell death during embryogenesis and inflammation
Source: Nat Commun. 2019 Sep 13;10:4157. doi: 10.1038/s41467-019-12033-8 (PMC6744441; doi:10.1038/s41467-019-12033-8)
Supplement: Supplementary file 3 — Reporting Summary [file 41467_2019_12033_MOESM3_ESM.pdf]

nature research

Corresponding author(s): DBPR-NCOMMS-18-34785A

Reporting Summary

Nature Research wishes to improve the reproducibility of the work that we publish. This form provides structure for consistency and transparency in reporting. For further information on Nature Research policies, see Authors & Referees and the Editorial Policy Checklist.

Statistical parameters

When statistical analyses are reported, confirm that the following items are present in the relevant location (e.g. figure legend, table legend, main text, or Methods section).

n/a	Confirmed	
☐	☒	The exact sample size (n) for each experimental group/condition, given as a discrete number and unit of measurement
☐	☒	An indication of whether measurements were taken from distinct samples or whether the same sample was measured repeatedly
☐	☒	The statistical test(s) used AND whether they are one- or two-sided<br>*Only common tests should be described solely by name; describe more complex techniques in the Methods section.*
☒	☐	A description of all covariates tested
☐	☒	A description of any assumptions or corrections, such as tests of normality and adjustment for multiple comparisons
☐	☒	A full description of the statistics including central tendency (e.g. means) or other basic estimates (e.g. regression coefficient) AND variation (e.g. standard deviation) or associated estimates of uncertainty (e.g. confidence intervals)
☐	☒	For null hypothesis testing, the test statistic (e.g. F, t, r) with confidence intervals, effect sizes, degrees of freedom and P value noted<br>*Give P values as exact values whenever suitable.*
☒	☐	For Bayesian analysis, information on the choice of priors and Markov chain Monte Carlo settings
☒	☐	For hierarchical and complex designs, identification of the appropriate level for tests and full reporting of outcomes
☒	☐	Estimates of effect sizes (e.g. Cohen's d, Pearson's r), indicating how they were calculated
☐	☒	Clearly defined error bars<br>*State explicitly what error bars represent (e.g. SD, SE, CI)*

Our web collection on statistics for biologists may be useful.

Software and code

Policy information about availability of computer code

Data collection	No software was used.
Data analysis	GraphPad Prism.

For manuscripts utilizing custom algorithms or software that are central to the research but not yet described in published literature, software must be made available to editors/reviewers upon request. We strongly encourage code deposition in a community repository (e.g. GitHub). See the Nature Research guidelines for submitting code & software for further information.

Data

Policy information about availability of data

All manuscripts must include a data availability statement. This statement should provide the following information, where applicable:

- Accession codes, unique identifiers, or web links for publicly available datasets
- A list of figures that have associated raw data
- A description of any restrictions on data availability

The authors declare that the data supporting the findings of this study are available within the paper. Source data for all figures are provided with the paper. And this statement is included in the manuscript.

Field-specific reporting

Please select the best fit for your research. If you are not sure, read the appropriate sections before making your selection.

☒ Life sciences ☐ Behavioural & social sciences ☐ Ecological, evolutionary & environmental sciences

For a reference copy of the document with all sections, see nature.com/authors/policies/ReportingSummary-flat.pdf

Life sciences study design

All studies must disclose on these points even when the disclosure is negative.

Sample size	The cell samples were collected and performed with at least three independent experiments. The samples from animal were collected from three mice every group and performed with three independent experiments. The human samples were independently collected for at least three times and measured with two duplicates. The exact size was described in the figure or the legend.
Data exclusions	N/A
Replication	The findings were reliably reproduced, and data shown are representative of three independent experiments.
Randomization	N/A. Experimental animals were grouped according to their genotypes and no randomization was necessary.
Blinding	N/A.

Reporting for specific materials, systems and methods

Materials & experimental systems

n/a	Involved in the study	
☒	☐	Unique biological materials
☐	☒	Antibodies
☐	☒	Eukaryotic cell lines
☒	☐	Palaeontology
☐	☒	Animals and other organisms
☒	☐	Human research participants

Methods

n/a	Involved in the study	
☒	☐	ChIP-seq
☐	☒	Flow cytometry
☒	☐	MRI-based neuroimaging

Antibodies

Antibodies used	Antibodies used in this study were described in the methods.
Validation	All antibodies used in this study were validated according to the manufacturer's instruction and all worked well.

Eukaryotic cell lines

Policy information about cell lines

Cell line source(s)	The source of HEK293T cells were from ATCC, and the source of immortalized MEFs were described in the methods.
Authentication	The HEK293T cells were purchased from ATCC and authenticated by the vendor, and the immortalized MEFs were authenticated according to the genotypes of embryos.
Mycoplasma contamination	All cell lines were tested to be negative for mycoplasma contamination.
Commonly misidentified lines (See ICLAC register)	No commonly misidentified cell lines were used.

Animals and other organisms

Policy information about studies involving animals; ARRIVE guidelines recommended for reporting animal research

Laboratory animals	Research animals were described in the methods.
Wild animals	The study did not involve wild animals.
Field-collected samples	The study did not involve samples collected from the field.

Flow Cytometry

Plots

Confirm that:

☒ The axis labels state the marker and fluorochrome used (e.g. CD4-FITC).

☒ The axis scales are clearly visible. Include numbers along axes only for bottom left plot of group (a 'group' is an analysis of identical markers).

☒ All plots are contour plots with outliers or pseudocolor plots.

☒ A numerical value for number of cells or percentage (with statistics) is provided.

Methodology

Sample preparation	For mice samples, the spleens from mice were perfused and digested into single-cell suspensions. After RBC lysis buffer treatment, the whole cells were washed with PBS and then stained with corresponding fluorescent antibodies and left at 4℃ until use. All mouse experiments were performed in compliance with institutional guidelines and according to the protocol approved by the Institutional Animal Care and Use Committee of Tsinghua University.
Instrument	For cell analysis, LSRFortessa (BD Biosciences) was used.
Software	Flow cytometry data were analyzed with FlowJo (Tree Star).
Cell population abundance	Without using sorting strategy.
Gating strategy	Characterization of Spleen T cells, B cells and Neutrophils.<br>In the spleen of mice, the living cell fractions gated from preliminary FSC/SSC gates could be further divided into four cell types: CD4+ T cells, CD8+ T cells, CD19+B220+ B cells and CD11b+Ly6G+ neutrophils.<br>The antibodies and fluorochrome used described as "Antibodies and reagents" in the methods.

☒ Tick this box to confirm that a figure exemplifying the gating strategy is provided in the Supplementary Information.
