# Supplementary material for: K63-linked ubiquitination regulates RIPK1 kinase activity to prevent cell death during embryogenesis and inflammation
Source: Nat Commun. 2019 Sep 13;10:4157. doi: 10.1038/s41467-019-12033-8 (PMC6744441; doi:10.1038/s41467-019-12033-8)

Figure 2d

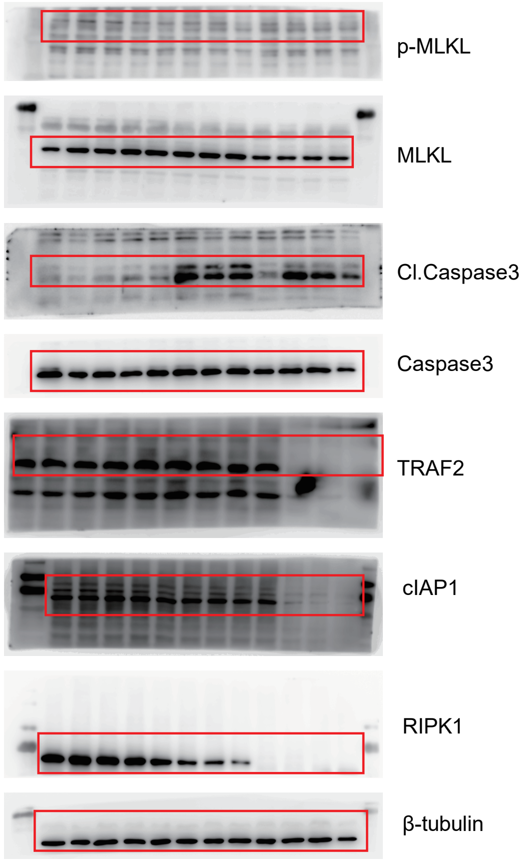

Figure 2e

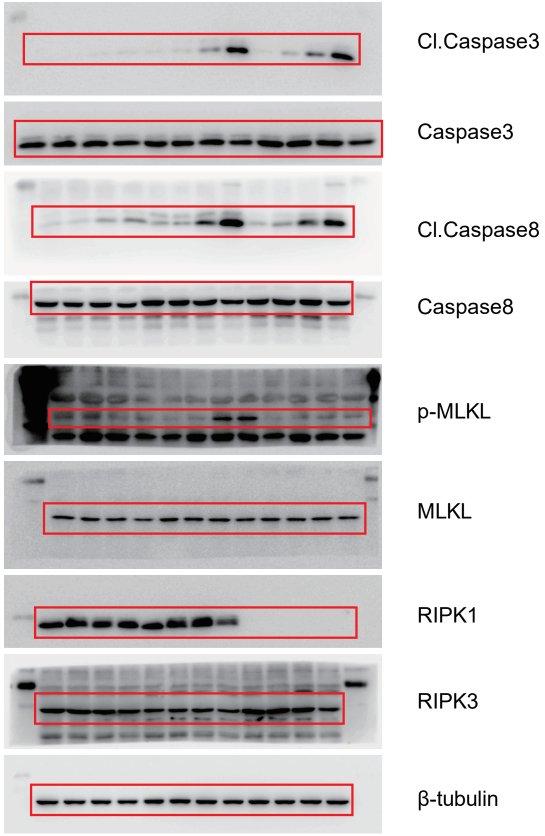

Figure 2f

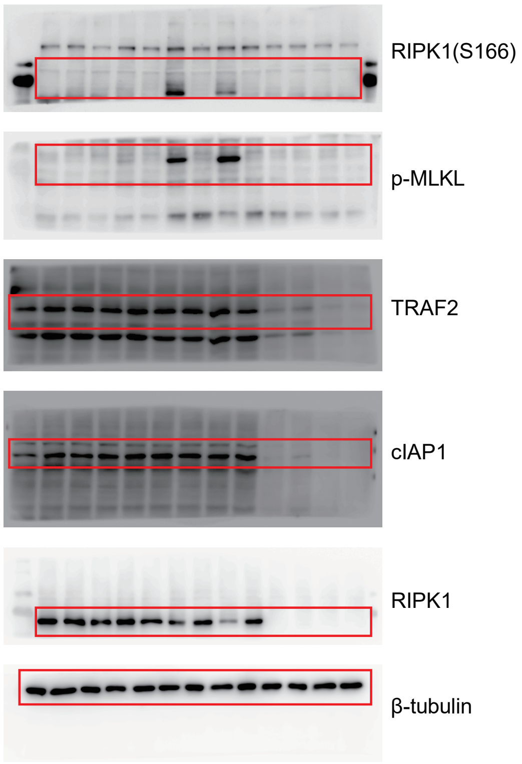

Figure 2g

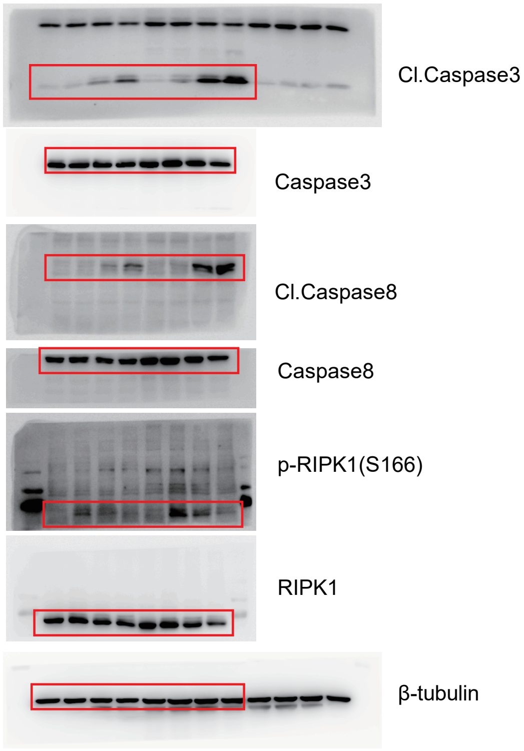

Figure 2h

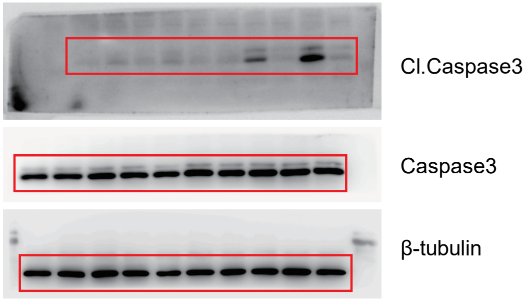

Figure 3a

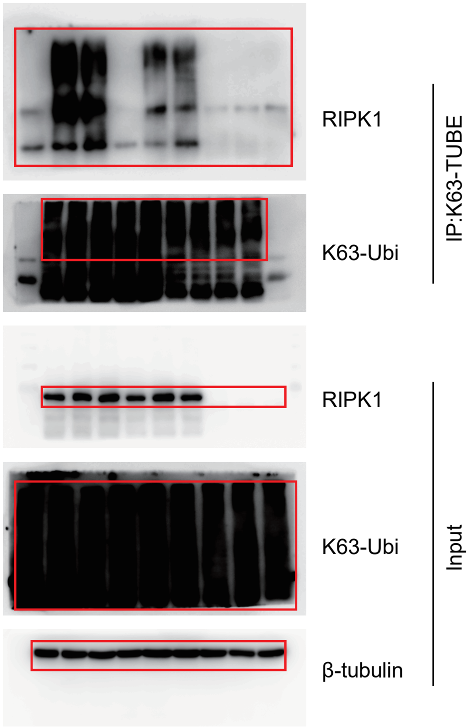

Figure 3b

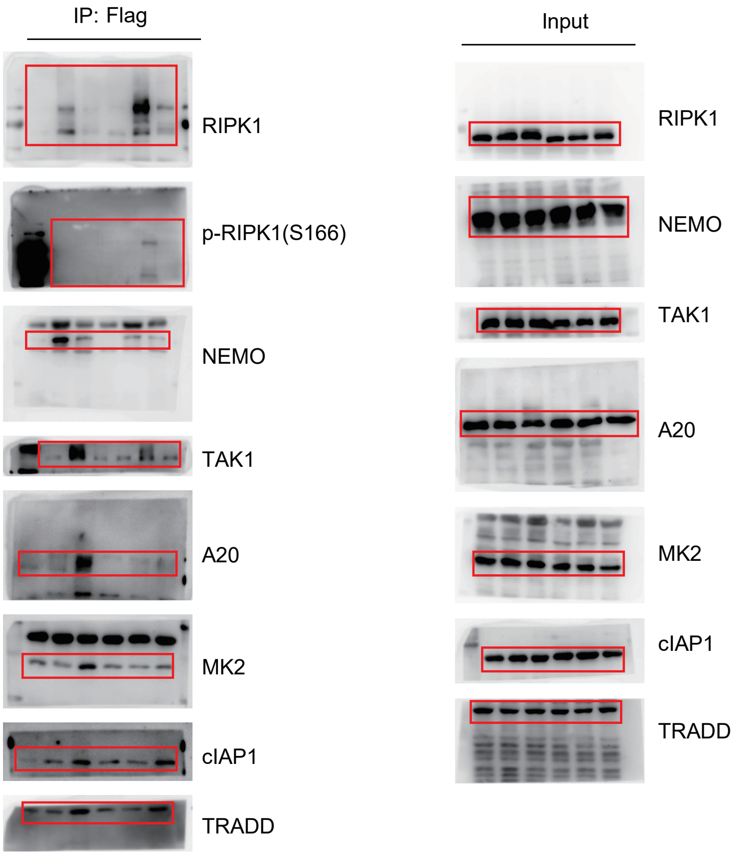

Figure 3c

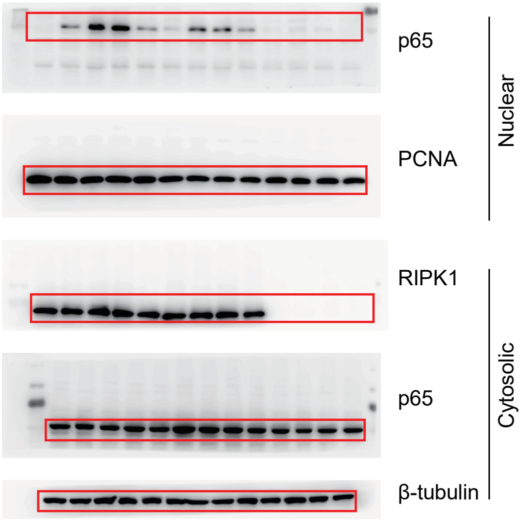

Figure 3e

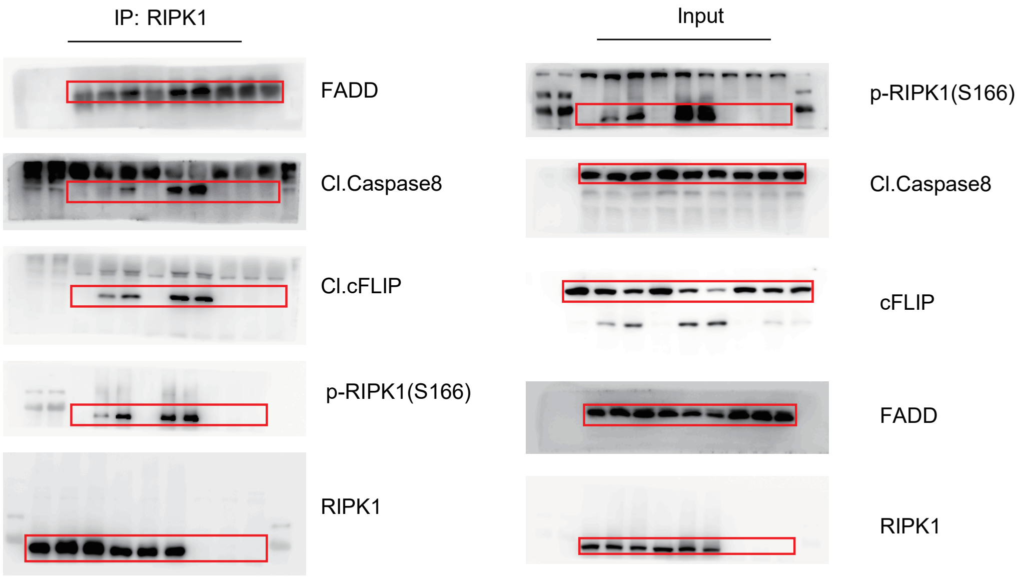

Figure 3f

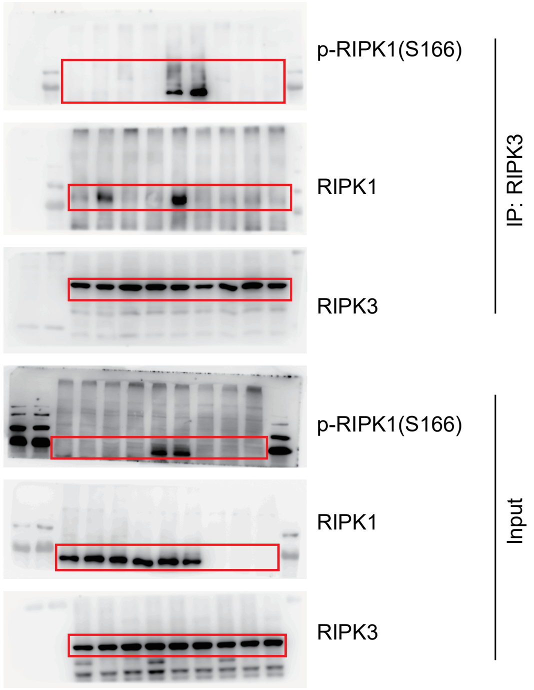

Figure 3g

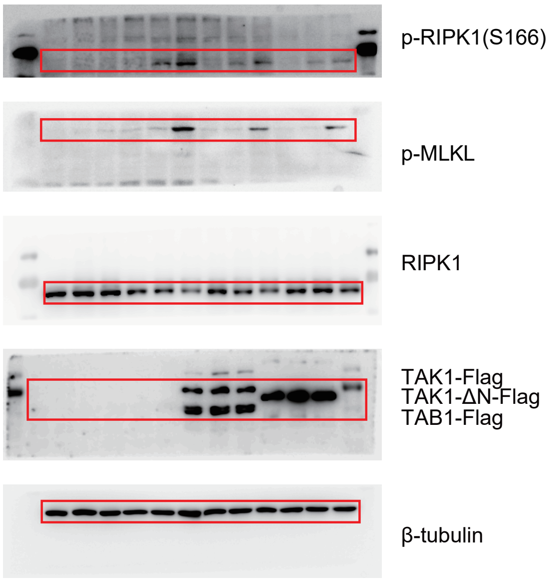

Figure 6h

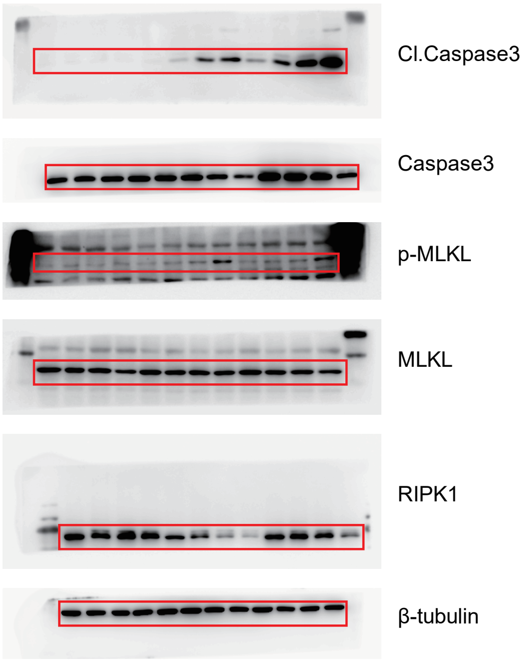

Figure 6i

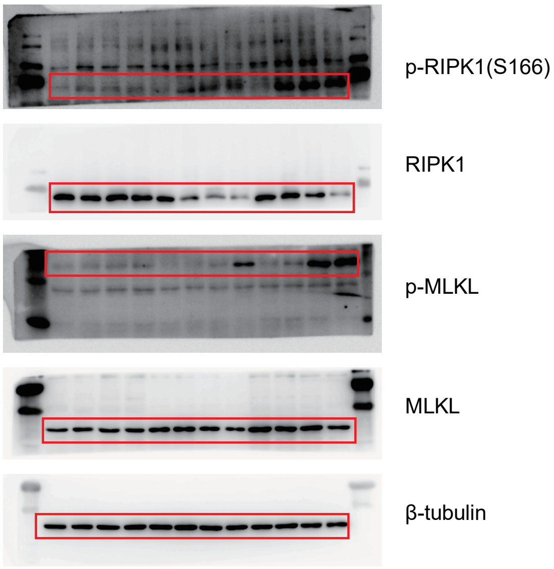

suppl. Fig 2a

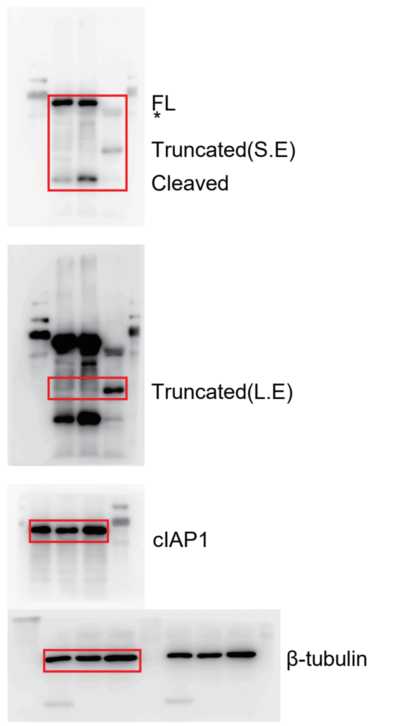

suppl. Fig 2b

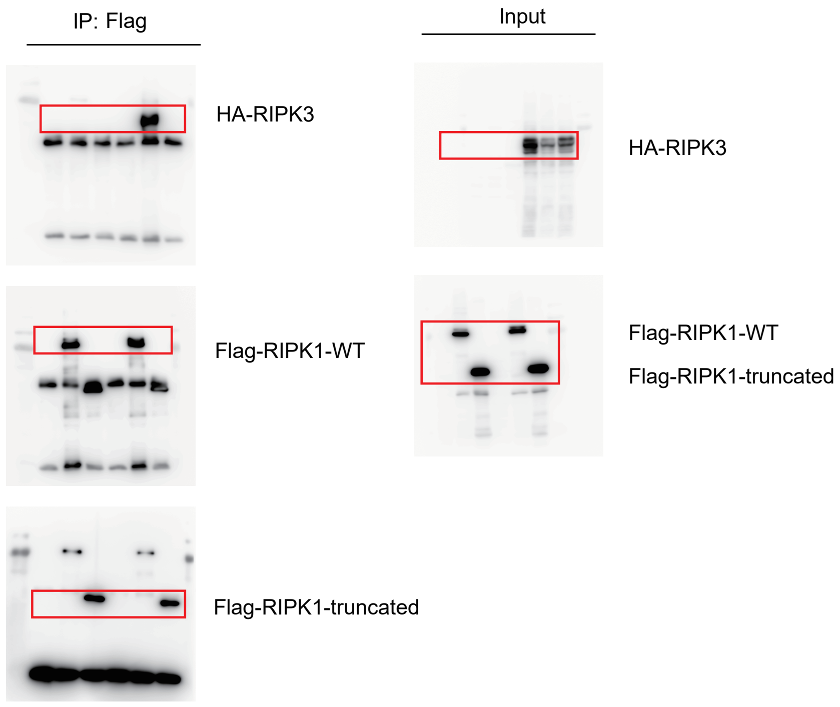

suppl. Fig 2d

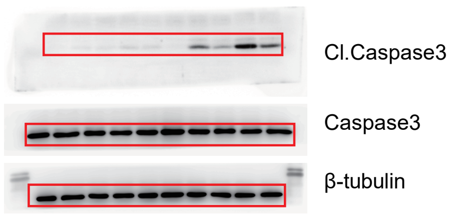

suppl. Fig 3a

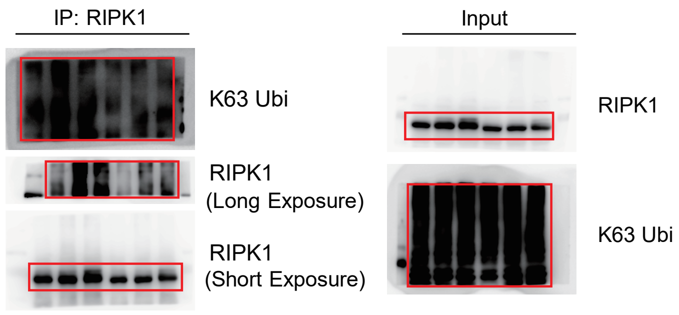

suppl. Fig 3b

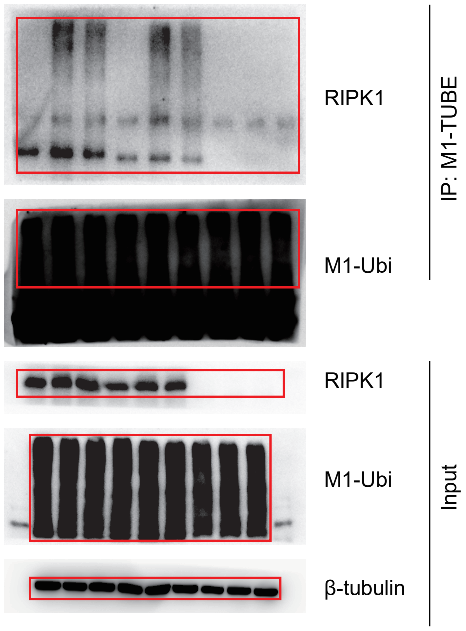

suppl. Fig 3d

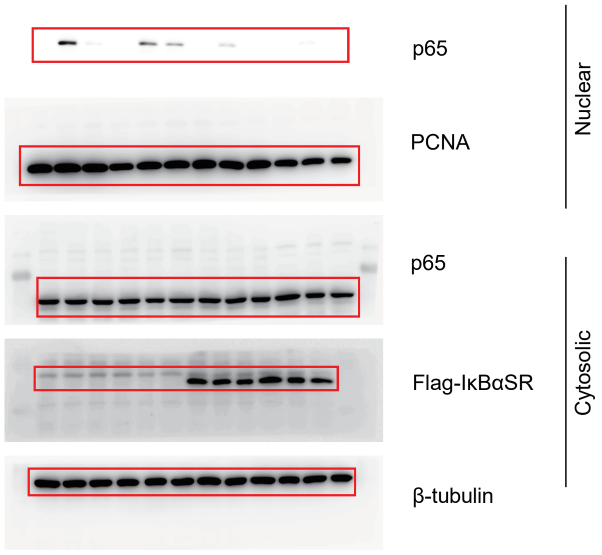

suppl. Fig 3e

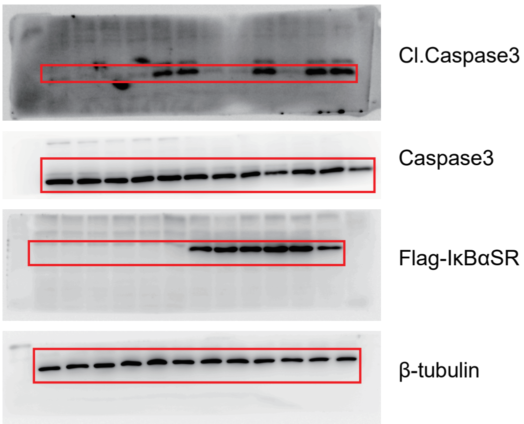

suppl. Fig 3g

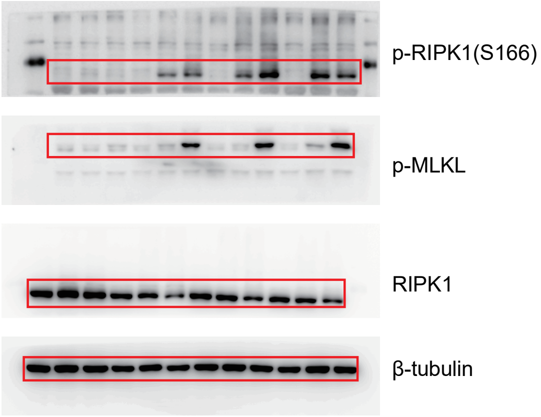

suppl. Fig 3i

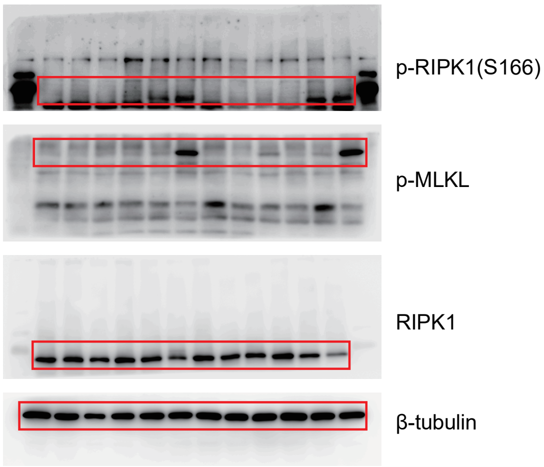

suppl. Fig 3f

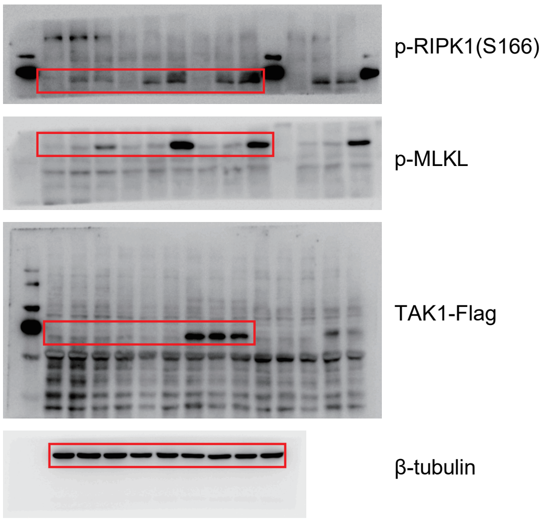

suppl. Fig 3h

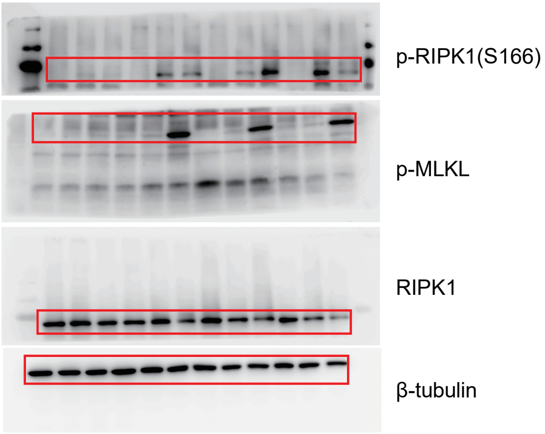

suppl. Fig 3j

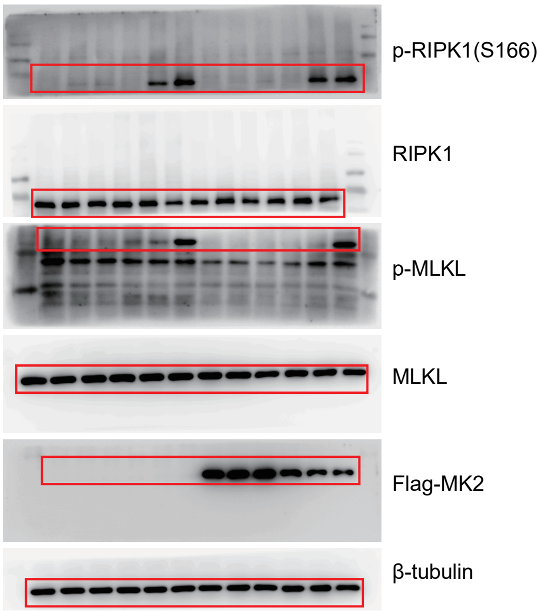

suppl. Fig 3k

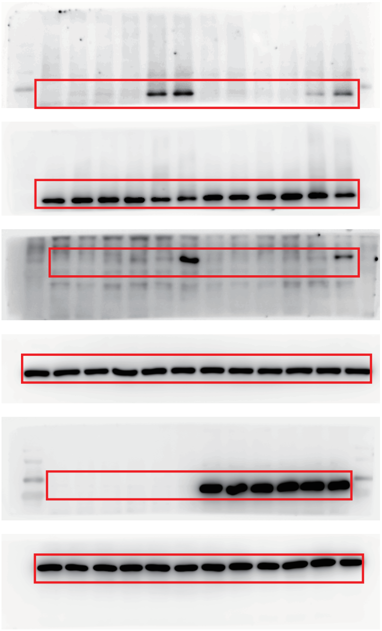

suppl. Fig 3l

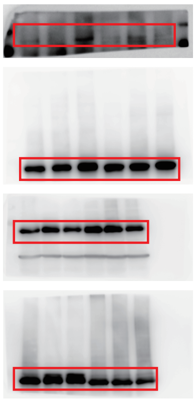

suppl. Fig 3m

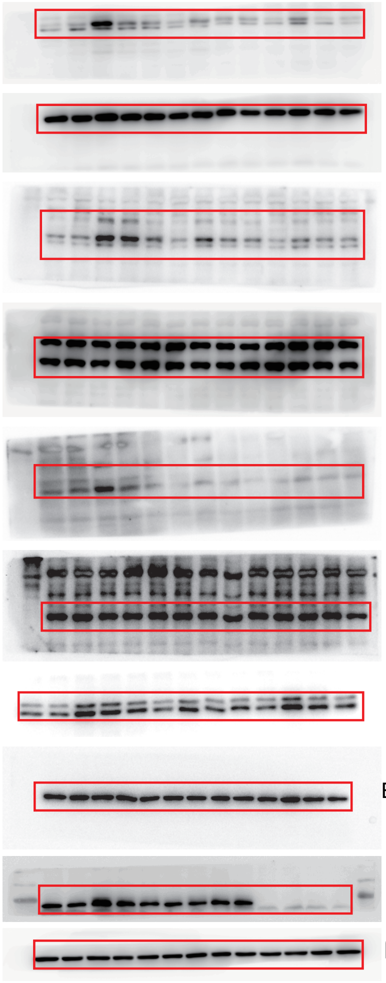

suppl. Fig 5a

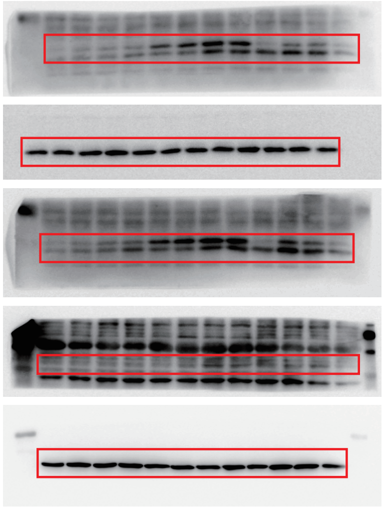

suppl. Fig 5b

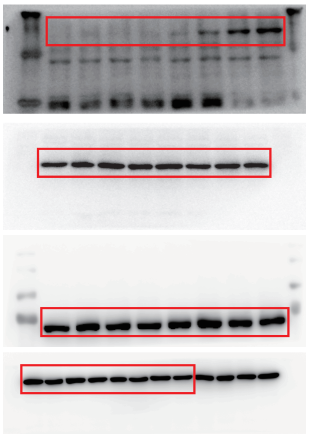

suppl. Fig 5c

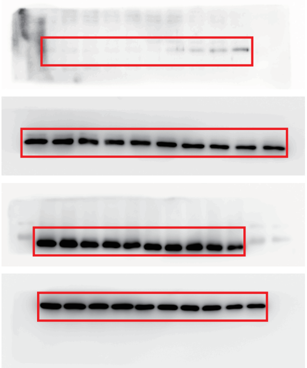

suppl. Fig 5d

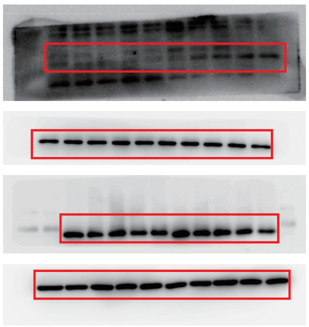

suppl. Fig 7i

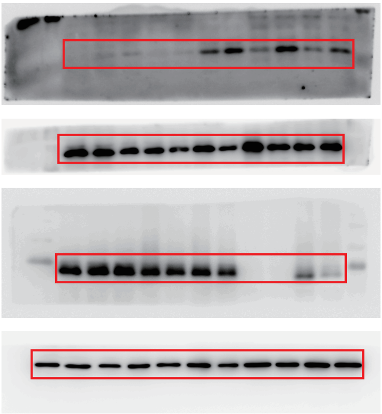

suppl. Fig 7j

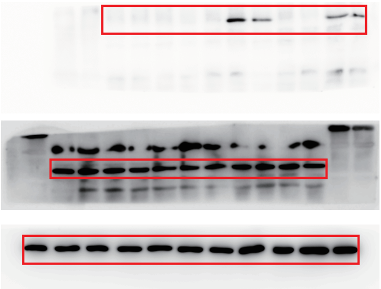

suppl. Fig 8b

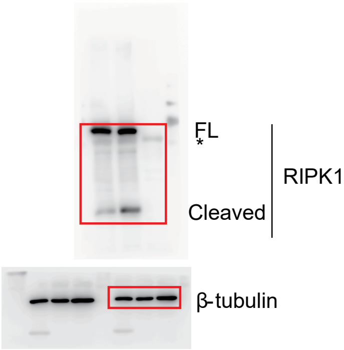

Supplement: Supplementary file 4 — Source Data [file 41467_2019_12033_MOESM4_ESM.zip › Source Data 2 - Uncropped versions of gels and blots presented in the figures.pdf]
